# Oxygen Vacancy Formation at Metal‒TiO₂ Interface Yielding Enhanced Photocatalytic Hydrogen Generation

**DOI:** 10.1002/advs.202501835

**Published:** 2025-06-20

**Authors:** Vien‐Duong Quach, Aparna Harsan, Maria Chiara Spadaro, Marc Botifoll, Jordi Arbiol, Marija Knezevic, Christophe Colbeau‐Justin, Franck Dumeignil, Hervé Vezin, Robert Wojcieszak, Tangui Le Bahers, Carine Michel, Mohamed Nawfal Ghazzal

**Affiliations:** ^1^ Institut de Chimie Physique Université Paris‐Saclay CNRS UMR 8000 Orsay F‐91405 France; ^2^ CNRS, ENS de Lyon, LCH – Laboratoire de Chimie UMR 5182 Lyon F‐69342 France; ^3^ Catalan Institute of Nanoscience and Nanotechnology (ICN2) CSIC and BIST Campus UAB Bellaterra Barcelona ES‐08193 Catalonia Spain; ^4^ ICREA Pg. Lluís Companys 23 Barcelona ES‐08010 Catalonia Spain; ^5^ LASIRE– Laboratoire Avancé de Spectroscopie pour les Interactions la Réactivité et l'Environnement Université de Lille CNRS UMR 8516 Lille F‐59000 France; ^6^ UCCS – Unité de Catalyse et Chimie du Solide Université de Lille CNRS UMR 8181 Lille F‐59000 France; ^7^ Department of Physics and Astronomy “Ettore Majorana” University of Catania and CNR‐IMM Via S. Sofia 64 Catania I‐95123 Italy; ^8^ Institut Universitaire de France 5 rue Descartes Paris F‐75005 France; ^9^ Ecole Normale Supérieure de Lyon CNRS, Laboratoire de Chimie UMR 5182 46 allée d’Italie Lyon F‐69364 France

**Keywords:** gold nanoparticles, oxygen vacancy, photocatalysis, strong metal‐support interaction, TiO_2_‐based photocatalyst

## Abstract

Strong Metal‐Support Interaction (SMSI) is a key concept in heterogeneous catalysis, but it remains underexplored in the context of photon‐to‐hydrogen conversion, as coupling of metallic nanoparticles with photocatalysts is overlooked and only discussed in terms of Schottky barrier formation. In this study, we provide deep insights into the effect of Au encapsulation with TiO_2_ overlayer on enhancing photocatalytic hydrogen generation. Our findings reveal that the construction of a SMSI‐like nanostructure induces the formation of oxygen vacancies at the Au‒TiO_2_ interface which actively facilitate charge carrier separation through interfacial band reconstruction. The presence of defects is evidenced by Electron Paramagnetic Resonance and X‐ray Photoelectron Spectroscopy, unveiling their relationship with photocatalytic activities. Consistent with experimental results, Density Functional Theory (DFT) calculations demonstrate that Au promotes oxygen vacancy formation. These vacancies located at the TiO_2_ surface significantly enhances H_2_O and MeOH adsorption during H_2_ evolution reactions. The SMSI‐like concept was extended to Pt, Pd, and Ag, in which the oxygen vacancy formation energy at the metal‐semiconductor interface varied depending on the metal, as computed by DFT. The results suggest that photocatalytic activity is related to the ease of oxygen vacancy formation, which is influenced by the nature of the metals.

## Graphical abstract

In this work, the oxygen vacancy formation is reported at the Au‐TiO_2_ interface in strong metal‐support interaction (SMSI) photocatalyst. The oxygen vacancies faciliate electron transfer and favor the adsorption of water and methanol at photocatalyst surface, thus enhancing the kinetic of photocatalytic hydrogen generation. The SMSI concept is extended to Pt, Pd, and Ag, in which the formation energy of oxygen vacancies at the metal‐semiconductor interface varied depending on the metal. The results indicate that the ease and the localization of oxygen vacancy formation is a critical factor governing photocatalytic acitivity.

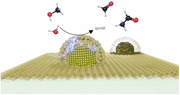

## 1 Introduction

The use of metal‐semiconductor composites represents an empirical approach for improving photocatalytic H_2_ production efficiency. In those such systems, metals can act either as a cocatalyst, collecting electrons from the semiconductor (SC),^[^ ^1^ ^]^ or as a photosensitizer, injecting electrons into the conduction band of SC and thereby harvesting visible light.^[^ ^2^, ^3^ ^]^ Charge carrier transport at metal‐semiconductor interface is governed by the band offset of the Schottky barrier.^[^ ^4^ ^]^ Extensive efforts have concentrated on tuning the electronic structure of the Mott–Schottky barrier by controlling the size of metal nanoparticles (NPs).^[^ ^5^ ^]^ In general, decorating SC with metal nanoparticles leads to an interface which occupies a small fraction of the total catalyst surface area, consequently restricting photocatalytic efficiency. A strategy to overcome this challenge involves increasing the interfacial surface area through the deposition of a thin semiconductor overlayer on the metal nanoparticles, a configuration indicative of the so‐called strong metal‐support interaction (SMSI).

The idea of a “Strong Metal‐Support Interaction” was introduced by Tauster et al.^[^ ^6^, ^7^ ^]^ in the 1970s; this concept has been frequently utilized by the heterogeneous catalysis community to design new catalysts and interpret catalytic activities. The SMSI is obtained, for instance, when a metal nanoparticle is covered by its support, generally a reducible metal oxide. The elaboration of interfaces with SMSI characteristics is particularly challenging. Indeed, the process necessarily involved high‐temperature reduction treatment.^[^ ^8^, ^9^ ^]^ To avoid such high‐temperature condition in SMSI‐based catalyst synthesis, other strategies have been proposed, such as mechanochemistry. A recent study employed a NaBH_4_‐assisted mechanochemical method under ambient conditions to generate Ti^3+^ and oxygen vacancies, which are crucial for SMSI encapsulation on Pd/anatase TiO_2_ catalysts.^[^ ^10^ ^]^ Our group proposed a soft‐chemistry approach to cover metal nanoparticles with TiO_2_, allowing us to obtain a variety of SMSI‐based TiO_2_ photocatalysts.^[^ ^11^, ^12^ ^]^ The method guarantees the formation of metastable anatase phase while avoiding the loss of dispersity and morphology of the noble metal nanoparticles.

In such systems, the interface between metal nanoparticles and semiconductor exhibited specific atomic and electronic structures, where oxygen vacancies were more likely to be formed, leading to a high impact on the chemical reactivity.^[^ ^5^, ^13^ ^]^ As an illustration, SMSI has recently been reported to enhance the catalytic performance of hydrogenation^[^ ^10^ ^]^ and H_2_O_2_ synthesis.^[^ ^14^, ^15^ ^]^ While SMSI‐based catalysts are widely studied in thermal catalysis, the concept remains comparatively underexplored in photocatalysis. Given that photocatalysts for H₂ production and CO₂ reduction typically compose of UV–Vis‐absorbing semiconductors decorated with metal nanoparticles, SMSI‐based nanostructures could play a key role in advancing this field. In our previous work, we reported an SMSI‐based photocatalyst in which Au nanoparticles (hereafter Au NPs) were encapsulated by a thin TiO₂ overlayer.^[^ ^11^ ^]^ This photocatalyst configuration exhibited higher efficiency in proton photoreduction for H_2_ evolution compared to those where Au NPs were merely deposited on the TiO_2_ surface. However, the mechanism underlying the difference in reactivity between these photocatalyst configurations leaves an open question as well as the possibility of extending this method to other metal nanoparticles. Investigating the effect of oxygen vacancy formation at the Au‐TiO_2_ interface could provide a rational explanation for the observed photocatalytic enhancement.^[^ ^16^, ^17^ ^]^ These vacancies – either formed during the catalytic cycle or present inherently at the interface – influence on the photocatalytic activities of TiO_2_.^[^ ^18^, ^19^, ^20^, ^21^, ^22^, ^23^, ^24^, ^25^ ^]^

In this study, we aim to elucidate the role of oxygen vacancies at the metal‐support interface in driving the superior photocatalytic activity of SMSI‐based photocatalysts. To do so, we revisited the model photocatalyst consisting of Au NPs deposited on a silica sphere and coated with a thin TiO_2_ overlayer, denoted as SMSI‐TiO_2_/Au. The SMSI configuration was compared to a conventional configuration in which TiO_2_ layer is first deposited on similar silica spheres, followed by the deposition of Au NPs on the top of TiO_2_ (referred to as Au/TiO_2_). For comparison, we also synthesized a metal‐free photocatalyst as a reference, named as SiO_2_@TiO_2_. These systems were thoroughly characterized using transmission electron microscopy (TEM), time‐resolved spectroscopy, and (photo)electrochemistry. The presence of defects was evidenced and discussed by Electron Paramagnetic Resonance (EPR) and X‐ray Photoelectron Spectroscopy (XPS) analyses to unveil the relationship with photo(electro)chemical properties. Density Functional Theory (DFT) calculations suggested a change in the oxygen vacancy formation energy at the metal‐TiO_2_ interface, depending on the different nature of metals. Based on these predictions, the SMSI was extended to other noble metals (Pt, Pd, and Ag), and a relationship between oxygen vacancy formation and photocatalytic H_2_ production is determined.

## 2 Results and Discussion

### 2.1 Synthesis and Structural Characterizations

**Figure 1:**
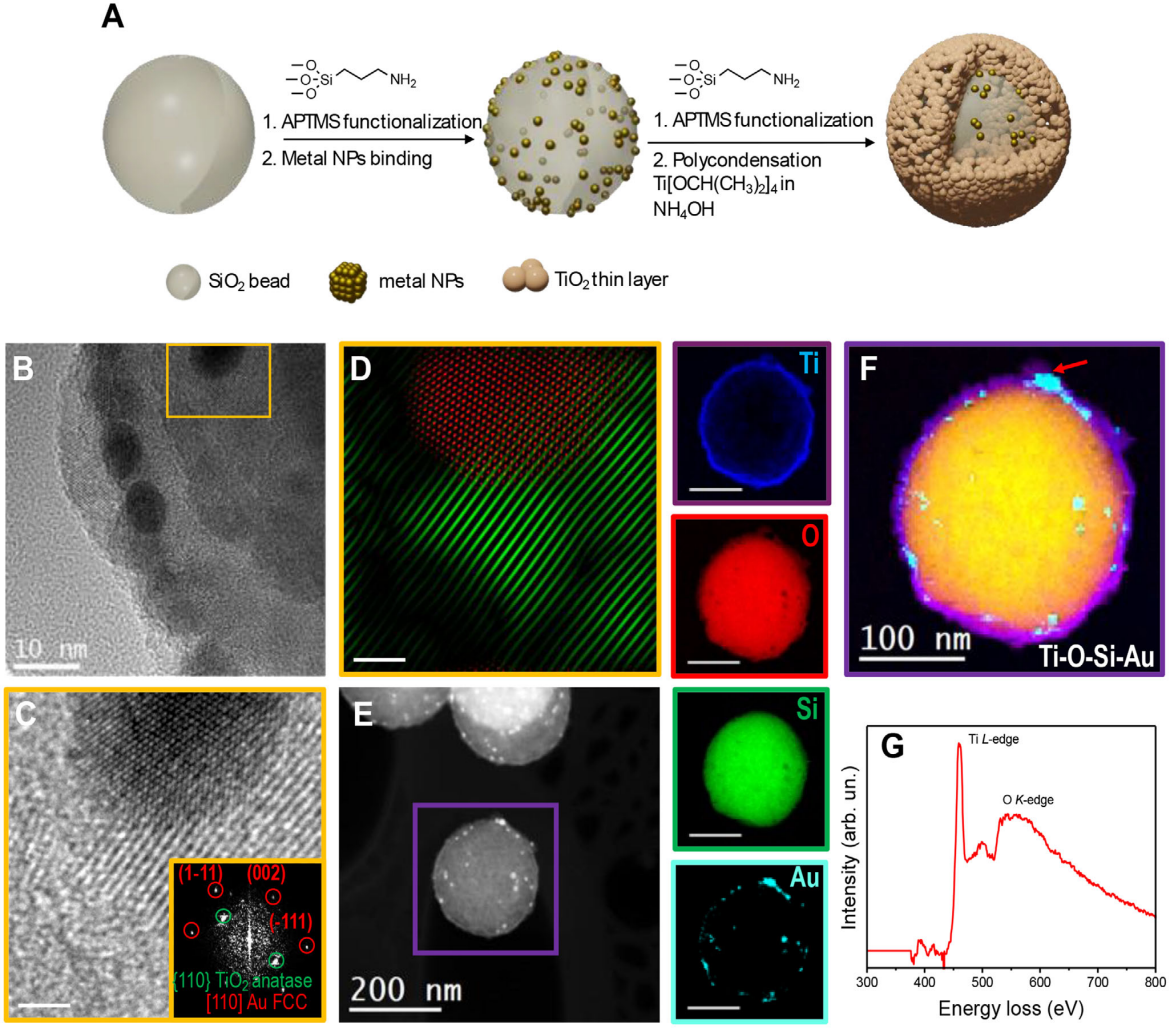
Schematic illustration of synthesis procedure and microscopic characterizations for SMSI photocatalyst. A) Soft‐chemistry synthesis pathway for SMSI‐TiO_2_/Au. B) HRTEM image of SMSI‐TiO_2_/Au. C) HRTEM of a localized Au nanoparticle and corresponding indexed power spectrum (inset). D) Frequency filtered map of an Au NP (red) and TiO_2_ (green) crystalline structure. E) STEM‐HAADF image of a SMSI‐TiO_2_/Au nanoparticle. F) STEM‐EELS mapping of separated elements and their combination: Ti L edge at 456 eV (ultramarine blue), O K edge at 532 eV (scarlet red), Si K edge at 1839 eV (kelly green), and Au M edge at 2206 eV (turquoise blue). G) EELS spectrum at the surface of an embedded Au nanoparticle (red arrow in (F)).

The SMSI photocatalyst was constructed by a soft chemistry method at room temperature (**Figure** **1**A; Figures S1 and S2, Supporting Information).^[^ ^2^ ^]^ SiO_2_ microspheres were used as cores which facilitate the homogenous distribution of Au NPs as well as the encapsulation by TiO_2_ overlayer. The bright field TEM and High Angle Annular Dark Field Scanning Transmission Electron Microscopy (HAADF‐STEM) images shown in Figures S3 and S4 (Supporting Information) provide an overview of the morphology of SMSI‐TiO_2_/Au and Au/TiO_2_, respectively. The images showed a homogenous size distribution of the spheres, which the average diameter is ≈250 nm. The size of both embedded and deposited Au NPs, estimated from HAADF‐STEM images, was in the range of 5–10 nm. Au NPs observed as bright spots in HAADF‐STEM images are well dispersed (Figure S3, Supporting Information). However, a bigger aggregate of Au NPs is found for Au/TiO_2_ than SMSI‐TiO_2_/Au (Figure S4, Supporting Information). In the latter, the encapsulation of Au NPs with TiO_2_ overlayer suppresses Ostwald ripening during the calcination step, hence limiting the NPs aggregation. Through High‐resolution TEM (HR‐TEM) and relative power spectra analysis, we detected in both configurations the crystal structures of Au NPs and TiO_2_, while SiO_2_ had an expected amorphous structure. The encapsulated Au NPs (diameter ≈6 nm, Figure 1B,C), in the SMSI configuration, exhibited a cubic FCC structure, oriented along its [110] zone axis. The power spectrum analysis (inset Figure 1C) indicated {110} atomic planes of TiO_2_ overlayer belonging to anatase phase. X‐ray Diffraction (XRD) analysis further confirmed the anatase phase of TiO_2_ and FCC structure of Au NPs for both configurations (Figure S6A, Supporting Information). This result is particularly appealing since the classical approach to construct SMSI through high‐temperature annealing in a reductive environment typically results in amorphous and/or TiO_2‐x_ sub‐stoichiometric structure.^[^ ^26^, ^27^ ^]^ The amorphous phase was reported as less active than anatase.^[^ ^28^ ^]^ The optical properties of the nanocomposites were analyzed by  Ultraviolet‐Visible Diffuse Reflectance Spectroscopy (UV‐Vis DRS) in the range of 200 to 800 nm (Figure S6B, Supporting Information). Au‐mediated samples exhibited absorption in both the UV and visible regions of the spectrum, whereas the Au‐free sample (SiO₂@TiO₂) showed absorption limited to the UV range. The Au/TiO₂ system demonstrated a broad absorption band centered ≈520 nm, characteristic of the localized surface plasmon resonance (LSPR) of Au NPs. In the case of the SMSI‐TiO₂/Au, this plasmonic band was red‐shifted by ≈70 nm. This shift can be attributed to an increase in the effective refractive index of the dielectric environment surrounding the Au NPs, resulting from encapsulation by the TiO₂ overlayer.^[^ ^2^, ^11^ ^]^ Specifically, in the Au/TiO₂ system, the Au NPs are partially exposed to air (n_air_ ≈ 1.0) and partially in contact with TiO₂ (n_TiO₂_ ≈ 2.1).^[^ ^29^ ^]^ In contrast, under SMSI conditions, as in the SMSI‐TiO₂/Au sample, the Au NPs are fully encapsulated by TiO₂ and also interact with the SiO₂ support (n_SiO₂_ ≈ 1.4).^[^ ^29^ ^]^ The observed redshift in the LSPR peak is, therefore, a direct consequence of the change in the local dielectric environment from air to higher‐index materials such as SiO₂. The STEM coupled with Electron Energy Loss spectroscopy (EELS) provided further morphological observation and chemical mapping of different elements (Ti, O, Si, and Au), hence visualizing their distribution within the nanostructures (Figure 1F). The chemical mapping indicated the core of the SMSI‐TiO_2_/Au mainly consisting of Si and O. Au NPs were covered by a TiO_2_ overlayer in SMSI‐TiO_2_/Au (Figure 1F). The EELS elemental distribution map obtained on the selected area containing Au NPs (red arrow, Figure 1F; Figure S5F, Supporting Information) further confirmed the formation of the TiO_2_ overlayer on the top of the Au NPs, assigned to Ti L edge at 456 eV and O K edge at 532 eV in the EELS spectrum of SMSI‐TiO_2_/Au (Figure 1G), but absent in the one of Au/TiO_2_ (Figure S5G, Supporting Information). In contrast to the SMSI structure, Au NPs were successfully deposited on TiO_2_ surface in Au/TiO_2_ photocatalyst. Additional structural and microscopic characterizations of this system are detailed and discussed in the supporting information (Figure S7, Supporting Information).

The Low‐energy Ion Scattering (LEIS) depth profiling spectroscopy provides further insights into the compositional depth of a surface (1–5 nm) by bombarding the sample with noble gas ions at a well‐defined and low energy of 4.1 × 10^15^ He^+^ ions per square centimeter (Figure S7B, Supporting Information). As the ion beam progressively etches the surface, gradients of concentrations from the upper to lower layers can be examined. The LEIS spectrum of Au/TiO_2_ appeared with four peaks at 2786 eV (Au), 2212 eV (Ti), 1715 eV (Si), and 1125 eV (O), demonstrating the existence of four elements on the surface until the depth of 10 nm. In the LEIS spectrum of SMSI‐TiO_2_/Au showed a vanishing of the Au peak, while the peaks of Si, Ti, and O were identical to the ones observed in the Au/TiO_2_ LEIS spectrum (Figure S7A, Supporting Information). The Si signal observed in both samples result from the diffusion of Si atoms in SiO_2_ core to the TiO_2_ layer during the calcination step at high temperature.^[^ ^30^, ^31^ ^]^ As shown in LEIS depth profiles (Figure S7B,C, Supporting Information), after 20 s of sputtering, the surface concentration of deposited Au in Au/TiO_2_ increased with the depth of He^+^ ion bombardment, whereas those of encapsulated Au in SMSI‐TiO_2_/Au generally stayed negligible, accompanied by the sharp incline of TiO_2_ signal. LEIS analysis directly confirms that a thin TiO_2_ overlayer fully encapsulates Au NPs in SMSI‐TiO_2_/Au, in agreement with HR‐TEM analysis.

### 2.2 Photocatalytic Hydrogen Production

**Figure 2:**
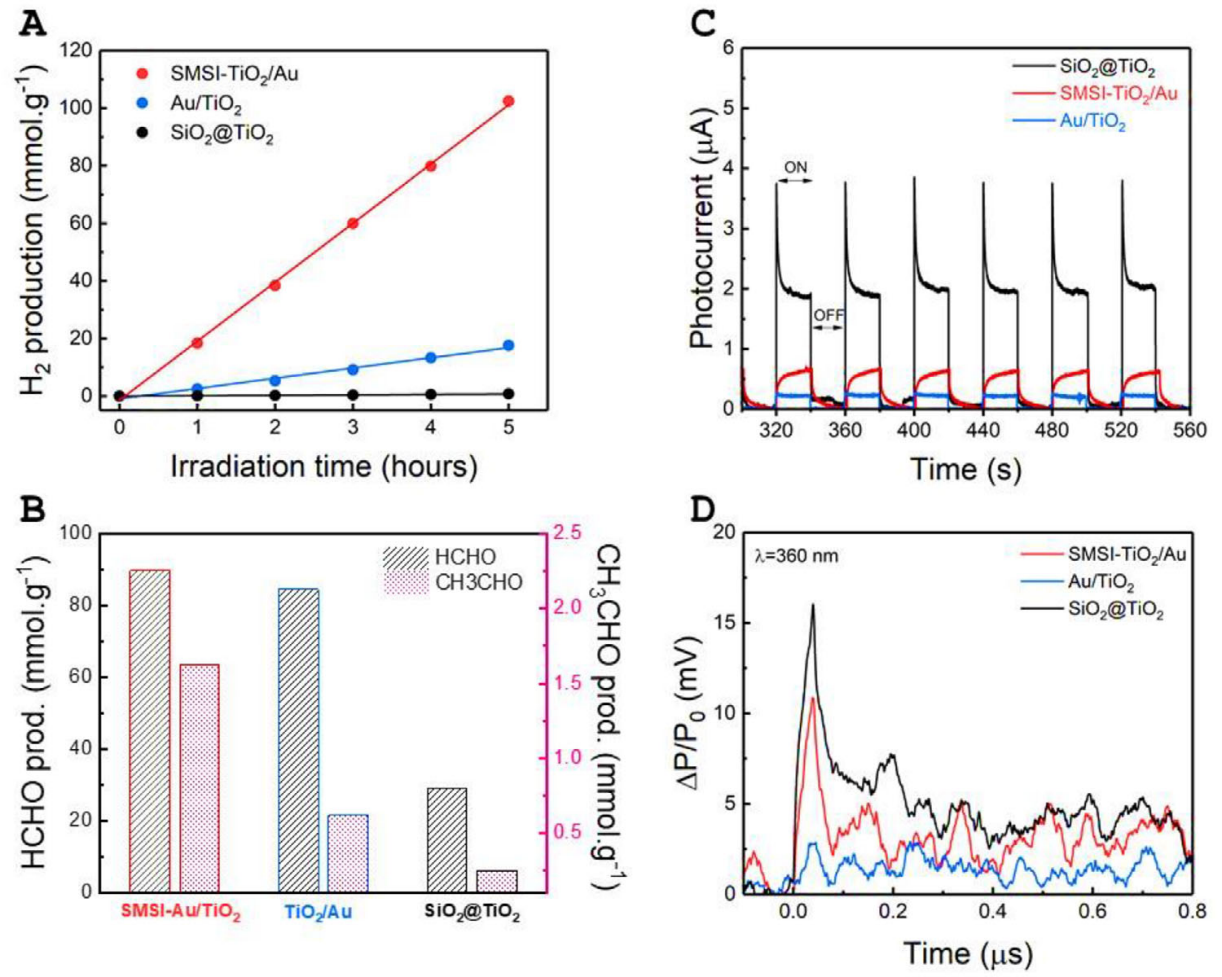
Photocatalytic evaluation of different TiO_2_‐based core@shell photocatalysts: A) Hydrogen production by SiO_2_@TiO_2_, SMSI‐TiO_2_/Au, and Au/TiO_2_. B) Formaldehyde and acetaldehyde production by SiO_2_@TiO_2_, SMSI‐TiO_2_/Au, and Au/TiO_2_. Charge carrier dynamics and lifetime characterization of SiO_2_@TiO_2_, SMSI‐TiO_2_/Au, and TiO_2_/Au: C) Amperometry *I–t* curves of the photoelectrodes at a fixed bias voltage 0.6 V vs. Ag/AgCl(saturated KCl) under intermittent AM 1.5G solar irradiation in K_2_SO_4_ 0.5M buffered pH = 7. D) Time‐resolved Microwave Conductivity (TRMC) signals of the samples triggered by laser irradiation (*I_ex_* = 1.396 mJ cm^−2^ at λ = 360 nm). E) Proposed photocatalytic mechanism on our core@shell nanostructures. Reaction conditions: 10 mg photocatalyst/10 mL solution, 25°C, H_2_O:CH_3_OH = 3:1 v/v, UV–Vis irradiation Mercury lamp 150W.

The photocatalytic performance of the photocatalysts was assessed by measuring the accumulated hydrogen production under UV–Vis illumination in an aqueous solution containing methanol as sacrificial electron donor (MeOH:H_2_O = 1:3 v/v). ICP‐OES (Inductively Coupled Plasma Optical Emission Spectrometry) analysis was utilized to determine the mass percentage of various elements (Si, Ti, O, Au) in our powder samples. Notably, the inert SiO_2_ component comprises ≈85% by weight, while the remaining active phases (TiO_2_ and Au), involved in photocatalytic reactions, constitute 15% by weight. Consequently, we adjusted the photocatalytic H_2_ evolution reaction (HER) rate based on the mass of the active phase. In **Figure** **2**A, Au‐free photocatalyst (SiO_2_@TiO_2_) registered the lowest photocatalytic activity for HER (≈0.15 mmol g^−1^ h^−1^). Coupling the photocatalyst with Au NPs (Au/TiO_2_) registered a hydrogen production that was 20 times higher, with a kinetic rate of ≈3.55 mmol g^−1^ h^−1^. The SMSI‐TiO_2_/Au photocatalyst remarkably increased the H_2_ production again, by almost one order of magnitude, up to ≈20.5 mmol g^−1^ h^−1^.

We also were interested in the oxidation reaction involving the methanol as sacrificial electron donor. This reaction was investigated by following the intermediates produced during photocatalytic reactions. After 5 h of illumination, reaction solutions were extracted from reactors and injected into a High‐performance Liquid Chromatography (HPLC). The results depicted in Figure 2B indicated that mainly HCHO and CH_3_CHO were produced, in which HCHO was the main product of photocatalytic methanol oxidation reactions. SMSI‐TiO_2_/Au demonstrated the capability to produce the highest amount of HCHO (≈90 mmol.g^−1^), followed by Au/TiO_2_ (≈85 mmol g^−1^), and to lesser extend SiO_2_@TiO_2_ (≈30 mmol.g^−1^). We have recorded the presence of CH_3_CHO, suggesting the possibility of C─C coupling, but a much lower amount compared to HCHO.^[^ ^32^, ^33^ ^]^ Similarly, SMSI‐TiO_2_/Au still outreached Au/TiO_2_ and SiO_2_@TiO_2_ in CH_3_CHO production yield. As a general trend, the methanol oxidation was more favorable on SMSI‐TiO_2_/Au rather than on Au/TiO_2_ and SiO_2_@TiO_2_ as it is for H_2_ production. Comparing different amount of the products produced after 5 h (Figure 2A,B), we can observe that all the three catalysts seem to be able to oxidize methanol while only the SMSI‐TiO_2_/Au is able to produce H_2_ efficiently. It is thus noteworthy to investigate the photoelectrochemical properties of these photocatalysts separately for oxidation and reduction reactions.

### 2.3 The Charge Carrier Dynamics

The charge carrier dynamics and lifetime were examined by photoelectrochemical (PEC) characterizations (Figure 2C) and Time Resolved Microwave Conductivity (TRMC) (Figure 2D), respectively. Amperometry transient photocurrent response was recorded under simulated solar light using an AM 1.5G 100 mW cm^−2^ at 0.6 V versus Ag/AgCl (saturated KCl) (Figure 2C). When the light is switched on, photogenerated electron‐hole pairs are separated rapidly, the holes migrate toward the interface between the semiconductor and electrolyte, while the electron is transferred to the fluorine‐doped tin oxide (FTO) electrode. Thus, the photo‐current cannot be limited here by the ability of the photocatalyst to perform the HER but it can be limited by its charge separation ability and/or by its efficiency in catalyzing the oxidation reaction that consumes the holes. The intensity of the *I–t* curve observed for SiO_2_@TiO_2_ is higher compared to that of SMSI‐TiO_2_/Au and Au/TiO_2_, pointing to higher number of photogenerated electrons flowing into the photoelectrochemical circuit in the SiO_2_@TiO_2_ case. In Au‐containing photocatalysts, the lower intensity of the photogenerated current could be due to the dynamics of the electron transfer at the Schottky junction between Au NPs and TiO_2_. This reduces the number of available electrons in the circuit under bias, resulting in lower current intensity for SMSI‐TiO_2_/Au and Au/TiO_2_. The shape of the *I*–*t* curves allows for assessing the efficiency of the charge carrier separation in each sample. A decay generally translates to charge carriers’ recombination or trapping of electrons and holes.^[^ ^34^ ^]^ The curves of SiO_2_@TiO_2_ exhibit a rapid decay of the signal pointing to fast charge carrier recombination. To lesser extent, the signal obtained for Au/TiO_2_ exhibits similar behavior. Conversely, an ascending photocurrent curve was recorded for SMSI‐TiO_2_/Au suggesting continuous charge accumulation during the light excitation of the photoelectrode. Encapsulated Au NPs likely act as electron reservoirs and reinjection from these Au NPs to TiO_2_ could take place through SMSI interface. On the contrary, Au NPs deposited on TiO_2_ surface (Au/TiO_2_) collect electrons but seems to not reinject them back into TiO_2_, reducing the current density. Photocurrent action spectra, encompassing Incident Photon‐to‐Current Efficiency (IPCE) and Absorbed Photon‐to‐Current Efficiency (APCE) under UV illumination (depicted in Figure S8A,B, Supporting Information, correspondingly), provide additional insight into the variation of photocurrent with wavelength at an applied bias of 0.6 V versus Ag/AgCl (≈1.23 V versus RHE, oxygen evolution reaction (OER) potential). These photon‐to‐current quantum efficiency measurements reveals that UV photons are optimal for photoelectrochemical water splitting on our materials. The highest conversion is obtained for the metal‐free sample, while SMSI‐TiO_2_/Au recorded APCE at over 1.25%. The Au/TiO_2_ photocatalyst observed negligible photon‐to‐current conversion (Figure S8B, Supporting Information). The low APCE observed for Au/TiO_2_ could be rationalized by shading effect due to Au NPs.

To examine the influence of SMSI encapsulation of Au NPs on the photocatalytic hydrogen evolution reaction, we carried out linear sweep voltammetry of the HER in K_2_SO_4_ electrolyte buffered at pH 7 under argon bubbling. Figure S8C (Supporting Information) illustrated that the cathodic current density was markedly elevated by SMSI construction in the photocatalytic system. The results demonstrate that the intrinsic HER activity can be significantly improved by covering Au NPs with TiO_2_ overlayer. The charge transport capability in the Au‐TiO_2_ interface was studied by Electrochemical Impedance Spectroscopy (EIS) (Figure S8D, Supporting Information). Nyquist plots were obtained from EIS measurements in both dark and under UV–Vis irradiation. The semicircle typically translating the charge transfer impedance between photoelectrode and electrolyte is observed to vary depending on the nature of the photocatalyst. SMSI‐TiO_2_/Au registered the lowest values of charge transfer resistance, as demonstrated by the smallest semicircle, compared to Au/TiO_2_. The result points to efficient charge transport in SMSI‐TiO_2_/Au, in agreement with the PEC results. However, since the photocurrent presented in Figure 2C is not the highest for SMSI‐TiO_2_/Au. We conclude that the charge‐transfer is not the only and the main phenomenon governing the photocurrent.

The charge carrier lifetime is one of the main effects in photocatalytic reduction reaction. More important than the carrier density, it is the long‐lived carriers that can perform photocatalytic reactions. We tracked the charge carrier mobility within nanostructures using Time‐resolved Microwave Conductivity (TRMC). Figure 2D shows the TRMC signal of three samples right after laser pulse excitation at 360 nm, whereby TiO_2_ is exclusively photoactivated. We observe that the overall TRMC intensity of the three signals follow the same order than the photo‐current presented in Figure 2C. Both SiO_2_@TiO_2_ and SMSI‐TiO_2_/Au catalysts experienced an intense TRMC signal. Both materials experience a first fast decay (before 0.1 µs) of similar characteristic time followed by a second (after 0.1 µs) slower decay, slightly faster for SMS‐TiO_2_/Au than for SiO_2_@TiO_2_. For Au/TiO_2_, we recorded a low intensity TRMC signal with fast decay, reflecting a very short lifetime of photogenerated charge carriers. As for SMSI‐TiO_2_/Au, the decay is attributed to the electrons being collected by Au NPs. In this case, although encapsulated Au NPs are expectedly electron scavengers, the electrons stored in Au NPs are likely to flow easily back into TiO_2_ crystalline structure through the strong metal‐support interaction regions over the Schottky barrier. This agrees with photoelectrochemical results. The excitation under visible light (λ = 420 and 550 nm) does not induce any TRMC signal for the Au‐free sample. However, longer TRMC signals were observed for gold‐containing samples (Figure S8E,F, Supporting Information). The visible light does not activate TiO_2_ (which only absorbs UV light as shown in Figure S6B, Supporting Information) but typically induces electrons at the surface of Au NPs that oscillate at the same frequency (localized surface plasmon resonance (LSPR)), followed by the injection of hot electrons from Au NPs to the conduction band of TiO_2_. The TRMC signal is remarkably higher for the SMSI‐TiO_2_/Au sample compared to Au/TiO_2_. This result supports the fact that the charge carrier dynamics is completely modified when changing the structure of the Au‐TiO_2_ junction, an aspect also observed for the photocurrent experiments.

From the reduction point of view, the electrochemical characterizations confirmed the larger ability of the SMSI‐TiO_2_/Au catalyst to perform the HER reaction efficiently. On the other side, from the oxidation viewpoint, the SiO_2_@TiO_2_ seems to be the best followed by SMSI‐TiO_2_/Au system. Altogether, to have an efficient photocatalyst, both oxidation and reduction reactions must be fast, this provides an electrochemical macroscopic explanation why SMSI‐TiO_2_/Au is so efficient as this photocatalyst is able to perform a fast oxidation reaction and a fast reduction reaction. This specific behavior of SMSI‐TiO_2_/Au is also observed in terms of charge carrier dynamics.

### 2.4 Oxygen Vacancies

Oxygen vacancies have been reported to be formed at the metal/oxide interface during the formation of strong metal‐support interaction and to play an important role in TiO_2_ photo‐catalytic activity.^[^ ^18^, ^19^, ^20^, ^21^, ^22^, ^23^, ^24^, ^25^ ^]^ Hereafter, we aim at exploring using experimental analysis and theoretical calculations the presence of oxygen vacancies and how they affect the photocatalytic efficiency of H_2_ generation in SMSI‐TiO_2_/Au sample.

**Figure 3:**
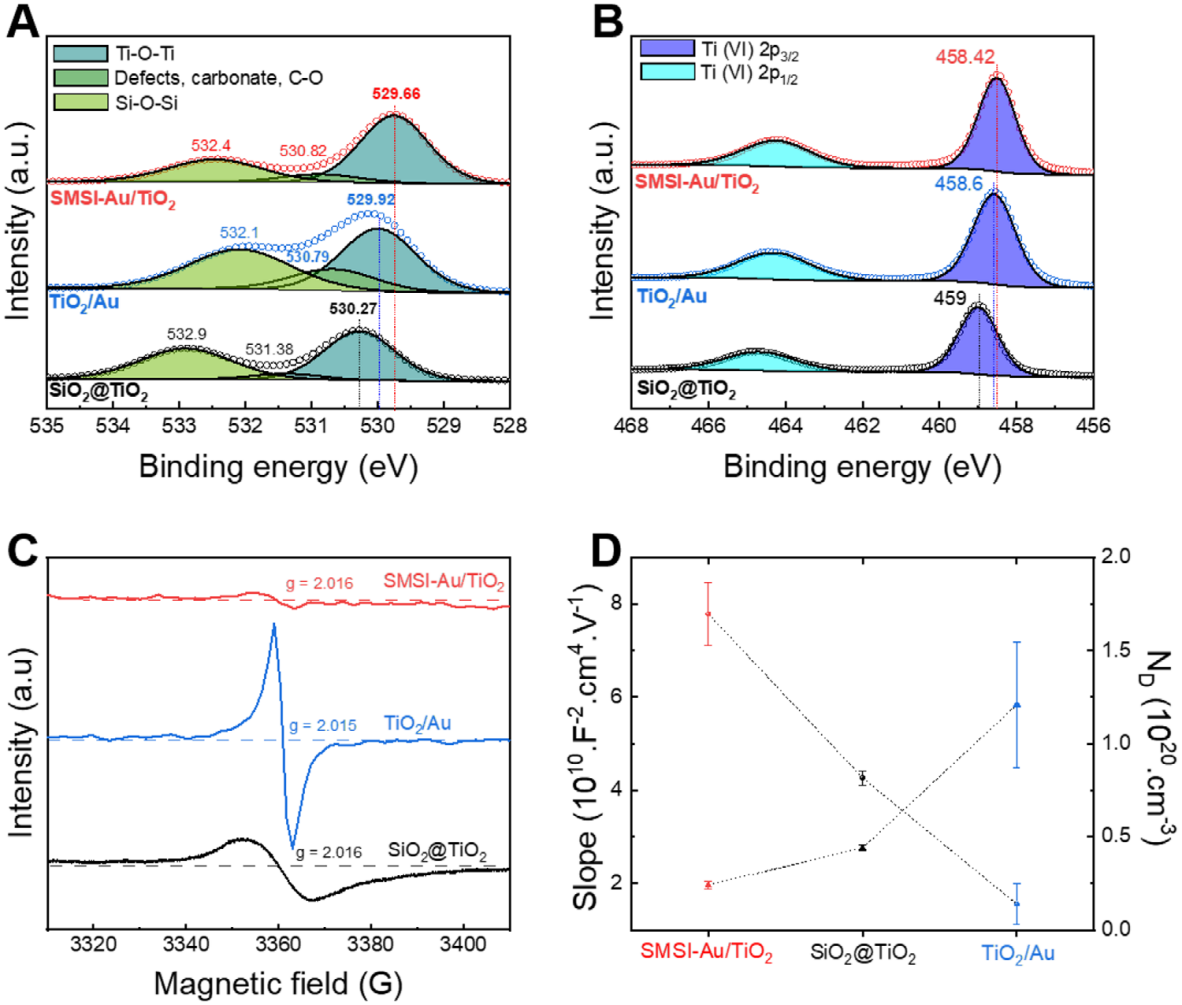
Surface and bulk characterizations for defect investigation: X‐ray photoelectron spectroscopy (XPS) spectra of A) O 1s and B) Ti 2p. C) Electron paramagnetic resonance (EPR) spectra. D) Donor (oxygen vacancy) density, N_D_, of the photocatalysts and its inverse proportion to the slope of Mott–Schottky plots.

Surface analysis of the photocatalysts was carried out by X‐ray Photoelectron Spectroscopy (XPS), outputting signals of core levels Au 4f, O 1s, and Ti 2p (Figure S9A,B, Supporting Information). High‐resolution XPS spectra of Au 4f show two main peaks at binding energy of 82.9 and 86.6 eV assigned to Au 4*f*_7/2_ and Au 4*f*_5/2_, respectively, for both Au‐containing samples (Figure S9C, Supporting Information). The Au 4f transition shifted toward lower binding energy, compared to previous reports,^[^ ^35^, ^36^ ^]^ indicating the formation of negatively‐charged *Au* ^δ −^ species in the samples. XPS is a surface‐sensitive technique that provides deep investigation of surface defects, particularly oxygen vacancy and Ti^3+^. The high‐resolution XPS spectra of O 1s (**Figure** **3**A) deconvoluted into three peaks, assigned to lattice oxygen in TiO_2_, hydroxyl groups, and oxygen in SiO_2_ core. The peaks of lattice oxygen in TiO_2_ showed a shift from 530.3 eV for SiO_2_@TiO_2_ to 529.9 eV for Au/TiO_2_ and further to 529.6 eV for SMSI‐TiO_2_/Au. The decrease of the binding energy of O 1s peak is attributed to the elimination of surface oxygen atoms,^[^ ^37^ ^]^ leading to the formation of oxygen vacancies. As a result of peeling off oxygen atoms, lone pairs of electrons remain, which can be captured by adjacent Ti^4+^ and subsequently lead to the formation of Ti^3+^ sites. The binding energy of Ti 2p_3/2_ peaks at 458.6 and 458.42 eV for Au/TiO_2_ and SMSI‐TiO_2_/Au, respectively, shifted to lower value by 0.4 and 0.6 eV compared to SiO_2_@TiO_2_ at 459 eV (Figure 3B). The binding energy of Ti^3+^ is lower compared to Ti^4+^, as reported by different studies.^[^ ^38^, ^39^ ^]^ Therefore, a decrease of the binding energy of the Ti2p XPS peak indicates the presence of Ti^3+^ on the surface. The density of oxygen vacancies and Ti^3+^ is then directly proportional to the magnitude of the Ti2p XPS peak shift to lower binding energy; in other words, a greater the negative shift observed for SMSI‐TiO_2_/Au translates in a higher density of surface defects.^[^ ^40^ ^]^ Therefore, the surface of the SMSI‐TiO_2_/Au sample should contain the largest density of surface oxygen vacancies, followed by the Au/TiO_2_ sample and the SiO_2_@TiO_2_ sample.

We employed Electron Paramagnetic Resonance (EPR) to probe defects generated in TiO_2_ bulk structure within our photocatalysts. We can expect that trapped electrons experience a strong EPR signal depending on their location into the sample. The EPR spectra in Figure 3C displayed an isotropic signal at a *g*‐factor of ≈2.015 for all samples, pointing to the presence of oxygen vacancies. The most intense signal was recorded for Au/TiO_2_, while the EPR spectra of SMSI‐TiO_2_/Au exhibited the slightest intensity. In contrast to XPS, which is a surface‐sensitive technique, the EPR signal presents mainly the bulk response. This indicates that the concentration of oxygen vacancies in the bulk of Au/TiO_2_ is greater than those of SiO_2_@TiO_2_ and SMSI‐TiO_2_/Au. Mott–Schottky analysis was performed to provide further confirmation of the presence of a large concentration of bulk oxygen vacancies in Au/TiO_2_ system. The positive slope of Mott–Schottky plots in Figure S9D (Supporting Information) represents an *n‐type* TiO_2_ in which oxygen vacancies donate electrons as major charge carriers. The carrier density estimated through the Mott–Schottky analysis typically refers to the bulk carrier concentration since it reflects the overall charge carrier behavior within the semiconductor.^[^ ^41^, ^42^ ^]^ Figure 3D shows a comparison between the charge carrier density among our photocatalysts. Au/TiO_2_ registered the highest density at 1.21  ×  10^20^ cm^−3^, followed by SiO_2_@TiO_2_ at 0.44  ×  10^20^ cm^−3^. The lowest density, at 0.24  ×  10^20^ cm^−3^, was estimated in bulk structure of SMSI‐TiO_2_/Au. The results obtained from the Mott‐Schottky analysis are in agreement with EPR analysis thus supporting the presence of bulk vacancies into the structures. Combining XPS, EPR, and Mott–Schottky experiments, it can be concluded that SMSI‐TiO_2_/Au photocatalyst has the largest concentration of surface oxygen vacancies, while the Au/TiO_2_ photocatalyst exhibit higher concentration of oxygen vacancies in bulk.

**Figure 4:**
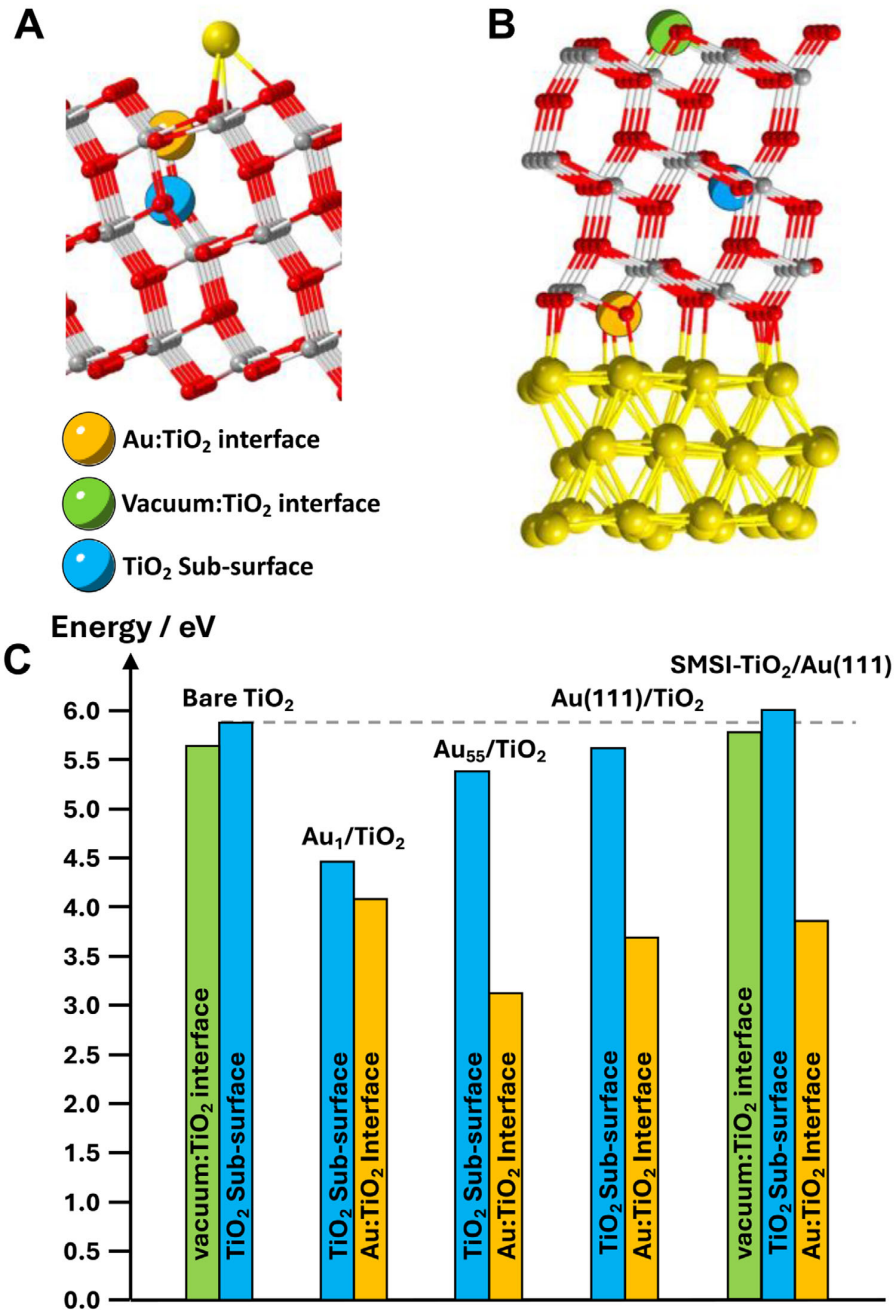
Structures and positions of oxygen vacancies for the A) Au1/TiO_2_ and B) SMSI‐TiO_2_/Au models. C) Computed oxygen vacancy formation energies for the different models and for different vacancy positions.

To understand why Au systematically promotes the formation of oxygen vacancies and what would be the preferential location of these vacancies that promotes the adsorption of water/ethanol molecules, we used Density Functional Theory (DFT) calculations to model the three photocatalysts. These calculations were performed using periodic boundary conditions along with the GGA‐PBE^[^ ^43^ ^]^ functional corrected by a Hubbard term for d‐electrons of Ti and using a split‐valence double zeta basis set.^[^ ^44^, ^45^ ^]^ More details can be found in Supporting Information Material. To assess the effect of the formation of oxygen vacancies (V_O_) on the surface reactivity, we computed the formation energy of oxygen vacancies on TiO_2_ slab in different scenarios, namely, bare TiO_2_ surface, TiO_2_ surface decorated by a single Au atom (Figure 3bisA), TiO_2_ surface decorated with Au NPs, TiO_2_ surface covered by Au monolayer (using TiO_2_ cell parameters). These three last systems are different models of Au/TiO_2_. We also tested Au surface covered by TiO_2_ overlayer (Figure 3bisB) (using Au cell parameters) as a model of SMSI‐TiO_2_/Au. For the sake of comparison with our experimental observations, we considered the main exposed surface (101) of TiO_2_ and (111) of Au. For each model, different positions of oxygen vacancies were considered. Subsequently, formation energies and the adsorption of MeOH and H_2_O were computed for V_O_ localized at the interface between Au and TiO_2_ (labeled as Au:TiO_2_ interface) or inside the TiO_2_ slab (labeled as TiO_2_ sub‐surface). The V_O_ formation energy on TiO_2_ surface in contact with vacuum was calculated, for a reference energy purpose. The data are summarized in **Figure** **4**C.

At first stage, the computed oxygen formation energies, ranging from 3.1 to 6.0 eV, are in agreement with the energies reported in the literature.^[^ ^46^ ^]^ Considering different positions of oxygen vacancies, these energies suggest that their formation at the TiO_2_ interface (either with vacuum or with gold) is easier compared to those at the sub‐surface, with a formation energy at the gold interface ≈3.7 eV and a formation energy at the sub‐surface position ≈5.8 eV. The V_O_ formation energy is also notably easier at the Au:TiO_2_ interface (≈3.7 eV) than at the vacuum interface (≈5.6 eV). The presence of Au NPs thus strongly promotes the V_O_ formation. The formation energies indicate that the oxygen vacancies are mainly localized at the Au‐TiO_2_ interface. Indeed, the XPS surface analysis indicates that in the Au/TiO_2_ system the 5 nm Au NPs screen the vacancies and their surrounding including Ti^3+^ while in the SMSI‐TiO_2_/Au system, the vacancies and Ti^3+^ nearby are only covered by a thin TiO_2_ layer of ≈1–2 nm thus detected in XPS.

The formation mechanism of V_O_ will be further discussed in the last section, when investigating the effect of the metal nature. However, here, the effect of V_O_ on surface reactivity was studied by calculating and comparing the adsorption energies of H_2_O and MeOH (to fit with experimental conditions) on different catalyst models, illustrated in Figure S13 (Supporting Information). These results highlight that the strongest adsorptions are obtained for the SMSI‐TiO_2_/Au model with an energy for H_2_O of −1.45 eV to be compared with −0.59 eV for the Au/TiO_2_ model and −0.91 eV for the SiO_2_@TiO_2_ model. These results point out the effects of oxygen vacancies, their location, and surrounding media on the capacity of the photocatalyst to adsorb molecules involved in the photocatalytic reactions. Indeed, the formation of oxygen vacancies at the Au‐TiO_2_ interface significantly facilitates the adsorption of H_2_O/MeOH, even at a site not directly connected to the vacancy. Combining the experimental and computational results converges to the fact that surface oxygen vacancies generated in the SMSI‐TiO_2_/Au photocatalyst probably play a key role in the high HER and methanol oxidation kinetics. Going to the full mechanism of methanol oxidation in these systems would be particularly helpful. However, only the grand‐canonical DFT can provide a reliable electrochemical reaction mechanism. This methodology is unfortunately too time and resource‐consuming now to be applied on complex interfaces as the ones investigated here.

### 2.5 Extending the SMSI‐Like Nanostructure to Different Metallic Nanoparticles

**Figure 5:**
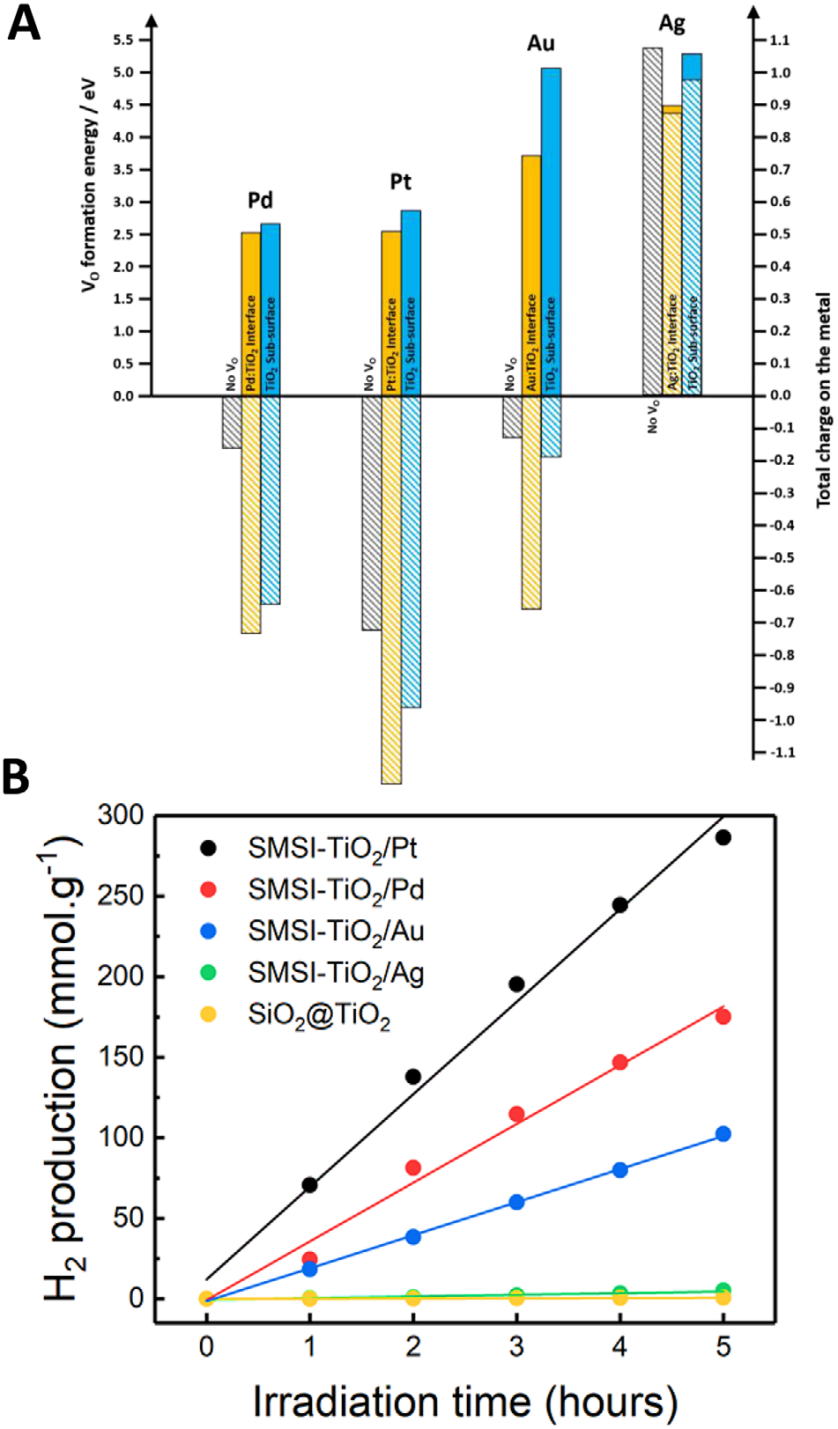
A) Computed oxygen formation energies (full bares) and integrated on the metal cluster Mulliken charges (dashed bares) as a function of the oxygen vacancy position and as a function of the metal for the SMSI‐TiO_2_/metal model. B) Photocatalytic hydrogen production for SMS‐like nanostructures, containing 4 different metals at reaction conditions: 10 mg photocatalyst/10 mL solution, 25°C, H_2_O:CH_3_OH = 3:1 v/v, UV–Vis irradiation from Mercury lamp 150W.

It is now necessary to test other metals and validate the relationship between oxygen vacancies and photocatalytic activity. We extended the gold‐based model to compute the formation energy of oxygen vacancies to other SMSI based‐nanostructures which Au was substituted with Pt, Pd, and Ag. In addition, Mulliken charge analysis was carried out to estimate the total charge on each metal before and after introducing oxygen vacancies, and the results are summarized in **Figure** **5**A. First, we found that the Pd, Pt, and Au metals are negatively charged upon TiO_2_ contact indicating an electron transfer from TiO_2_ to the metal. The opposite is computed for Ag. This follows the variation of the work function that is notably high for Pt (5.1–5.9 eV), Pd (5.2–5.6 eV), and Au (5.3–5.5 eV) and lower for Ag (4.5–4.7 eV).

Metals were more negatively charged in the presence of oxygen vacancies, which is attributed to the removal of oxygen atoms that create excessive electrons that are then transferred to the metal matrix. In the case of silver, the metallic charge remains positive but with a lower value. When oxygen vacancies are located at the metal:TiO_2_ interface, Pt was found to be the most negatively charged (−0.96), followed by Pd (−0.64) and Au (−0.19), whereas computational calculations output a positive value of charge on Ag (0.97). The computed oxygen vacancies formation energies suggest that the oxygen vacancies are more likely to be generated at the metal‐TiO_2_ interfaces than on a free surface (Figure 5A). As for the charges, there is a trend between the metals, the formation energy is lower for Pt and Pd than for Au, and the formation is the largest for Ag. The capacity of the metal to capture the electron released by the formation of the oxygen vacancy is probably an explanation of the low oxygen formation energy in Pt, Pd, and Au metals. The formation of oxygen vacancies could be then induced by electronic rearrangements at the Au NPs and TiO_2_ interface. Such charge redistribution is reported to be typically confined to a few atomic layers adjacent to the Au‐O_v_‐TiO_2‐x_ interface and is accompanied by changes in the oxidation states of either the Au NPs or the metal cations in TiO_2_.^[^ ^47^ ^]^ The formation mechanism of oxygen vacancies at the vicinity of the metal/support interface is in agreement with reported studies and the DFT modeling presented below.^[^ ^48^, ^49^ ^]^

Experimentally, we synthesized different SMSI‐based nanostructures by replacing Au nanoparticles with different metallic nanoparticles, including Pt, Pd, and Ag. In Figure 5B, SMSI‐TiO_2_/Pt catalyst provides the highest photocatalytic H_2_ production rate followed by SMSI‐TiO_2_/Pd and SMSI‐TiO_2_/Au. Compared to these samples, SMSI‐TiO_2_/Ag showed the lowest and almost negligeable HER rate. The shape of transient photocurrent curves in Figure S14B (Supporting Information) reveal the different characteristics of charge carrier transfer within our photocatalysts. SMSI‐TiO_2_/(Pt, Pd, Au) recorded continuous uphill photocurrent response, while the *I–t* curve of SMSI‐TiO_2_/Ag became flat after reaching a spike shortly, suggesting that the electronic structure of the metal–TiO_2_ interface is notably different between SMSI‐TiO_2_/(Pt, Pd, Au) on one side and SMSI‐TiO_2_/Ag on the other side. Cathodic linear sweep voltammetry additionally show the higher current density and smaller onset potential in order of SMSI‐TiO_2_/(Pt, Pd, Au, and Ag) (Figure S14C, Supporting Information). These results are supported by the DFT computed charges of the metal NPs presented above. Pt, Pd, and Au NPs were computed to be negatively charged on TiO_2_ surface while Ag NP is computed to be positively charged. This would suggest a rectifying behavior of the *I–V* curve for SMSI‐TiO_2_/(Pt, Pd, Au) catalysts and a Ohmic contact for SMSI‐TiO_2_/Ag catalyst. It means that for SMSI‐TiO_2_/(Pt, Pd, Au) catalysts, upon an increase of conduction band electron density (by light absorption for instance) the electron transfer toward the metal NP is promoted while the reverse electron transfer is blocked by the Schottky barrier leading to an accumulation of electron in the metal NPs. On the opposite, for SMSI‐TiO_2_/Ag, the electron transfer is possible between the metal NP and the TiO_2_ (forward and backward) leading to less efficient charge carrier separation. While interesting, we aim to underline that this interpretation must be taken with caution since it is not necessarily valid for systems where the semiconductor is just a nanometre scale layer and the metal a nanometre scale particle. Time‐resolved microwave conductivity was subsequently performed to examine the mobility of major charge carriers (electrons), which were generated by laser pulses at λ = 360, 420, and 550 nm. In Figure S14E (Supporting Information), upon UV excitation (thus involving the TiO_2_ absorption), SMSI‐TiO_2_/(Pt, Pd) catalysts experience no intense first signal but a slight increase of the TRMC signal. The results suggested that there is a fast electron transfer from TiO_2_ to Pt/Pd during the laser pulse (<8 ns) followed by a slow release of the electron into TiO_2_ explaining the plateau for Pd and Pt metals. The TRMC signal of SMSI‐TiO_2_/Ag neither do not show any first intense peak but rather a decrease of the electron density that could support the idea of a catalyst facilitating the charge carrier recombination. The SMSI‐TiO_2_/Au catalyst has a unique behavior. After a first intense peak, the signal slowly decreases in intensity like for SMSI‐TiO_2_/Ag. Figure S14F (Supporting Information) displays TRMC signals triggered by a laser excitation in the visible part of the spectrum thus TiO_2_ absorption is not involved. In that case, these signals remain constant indicating that the charge carriers’ photo‐generated by the light absorption in metal NPs are notably stable (UV–Vis absorption spectra of the catalysts in Figure S14G, Supporting Information).

Beyond to charge carrier dynamics, as we mentioned previously, oxygen vacancies could play a key role for the enhanced photoactivity of SMSI‐TiO_2_/Pd and SMSI‐TiO_2_/Pt. As presented above for the Au‐based catalysts, we estimated the concentration of the charge carrier densities generated by the oxygen vacancies from the slope of Mott–Schottky plots for all the metals considered in this section (Figure S14H, Supporting Information). SMSI‐TiO_2_/(Pd,Pt) possess the highest defect density, above 2.0 × 10^20^ cm^−3^ for both metals This density significantly decreases to 0.24 × 10^20^ cm^−3^ and 0.11  ×  10^20^ cm^−3^ for SMSI‐TiO_2_/Au and SMSI‐TiO_2_/Ag, respectively. This experimental output rationally follows the prediction of interfacial oxygen vacancy formation energy in the DFT section (Figure S14A, Supporting Information; Figure 4A).

It is noteworthy that depending on the characterization technique, the SMSI‐TiO_2_/Au catalyst experience sometimes the behavior of the SMSI‐TiO_2_/(Pt, Pd) catalyst and sometimes the one of SMSI‐TiO_2_/Ag. It is also valid for DFT calculations. As a matter of fact, the computed charge of Au NP while negative is quite small in absolute value (compared to the Pt and Pd metals). It subsequently supports the idea that Au is more an intermediate metal between Pt and Pd in one side and Ag on the other side. This is also observed for the photocatalytic activities.

## 3 Conclusion

An SMSI‐like nanostructure was obtained by encapsulating Au nanoparticles within a TiO_2_ overlayer. The LEIS surface analysis and the morphological characterization, along with chemical mapping, confirmed the successful encapsulation of Au nanoparticles, constructing SMSI‐like interface. This encapsulation resulted in an impressive enhancement of the photocatalytic H_2_ generation. Oxygen vacancies, whose formation is facilitated by the Au‐TiO_2_ interface, are probably responsible of the enhanced photocatalytic efficiency. We found that the oxygen vacancies improve the adsorption/desorption energy of MeOH and H_2_O and the photogenerated charge carrier lifetime. Building on this concept, we extended the SMSI‐like concept to Pt, Pd, and Ag, which exhibited variable oxygen energy formation at the metal‐TiO_2_ interface. The nature of the metal nanoparticles significantly influenced the energy formation of oxygen vacancies. High work function metals, such as Pt, Pd, and Au, markedly lowered the oxygen vacancy formation energy. In contrast, lower work function metals like Ag exhibited an opposite trend. A clear correlation was uncovered between the ease of oxygen vacancy formation and photocatalytic H_2_ generation. These findings highlight the nuanced interplay between metal‐support interactions and photocatalytic mechanisms, offering strategic insights for the rational design of advanced photocatalytic systems.

## Conflict of Interest

The authors declare no conflict of interest.

## Supporting information



Supporting Information

## Data Availability

The data that support the findings of this study are available from the corresponding author upon reasonable request.
