# Supplementary material for: Oxygen Vacancy Formation at Metal‒TiO₂ Interface Yielding Enhanced Photocatalytic Hydrogen Generation
Source: Adv Sci (Weinh). 2025 Jun 20;12(32):e01835. doi: 10.1002/advs.202501835 (PMC12407321; doi:10.1002/advs.202501835)
Supplement: Supplementary file 1 — Supporting Information [file ADVS-12-e01835-s001.pdf]

ADVANCED SCIENCE
Open Access

Supporting Information

for *Adv. Sci.*, DOI 10.1002/advs.202501835

Oxygen Vacancy Formation at Metal–TiO_2 Interface Yielding Enhanced Photocatalytic Hydrogen Generation

*Vien-Duong Quach, Aparna Harsan, Maria Chiara Spadaro, Marc Botifoll, Jordi Arbiol, Marija Knezevic, Christophe Colbeau-Justin, Franck Dumeignil, Hervé Vezin, Robert Wojcieszak, Tangui Le Bahers, Carine Michel and Mohamed Nawfal Ghazzal**

Supporting Information

Oxygen vacancy formation at metal–TiO_2 interface yielding enhanced photocatalytic hydrogen generation

Vien-Duong Quach,[a] Aparna Harsan,[g] Maria Chiara Spadaro,[b,c] Marc Botifoll,[b] Jordi Arbiol,[b,d] Marija Knezevic,[a] Christophe Colbeau-Justin,[a] Franck Dumeignil,[e] Hervé Vezin,[f] Robert Wojcieszak,[e] Tangui Le Bahers,[g,h] Carine Michel,[g] Mohamed Nawfal Ghazzal.[a**]

[a.] *Institut de Chimie Physique, Université Paris-Saclay, CNRS UMR 8000, F-91405 Orsay, France*

[b.] *Catalan Institute of Nanoscience and Nanotechnology (ICN2), CSIC and BIST, Campus UAB, Bellaterra, ES-08193 Barcelona, Catalonia, Spain*

[c.] *Department of Physics and Astronomy "Ettore Majorana", University of Catania and CNR-IMM, Via S. Sofia 64, 95123 Catania, Italy*

[d.] *ICREA, Pg. Lluís Companys 23, ES-08010 Barcelona, Catalonia, Spain*

[e.] *UCCS – Unité de Catalyse et Chimie du Solide, Université de Lille, CNRS UMR 8181, F-59000 Lille, France*

[f.] *LASIRE – Laboratoire Avancé de Spectroscopie pour les Interactions la Réactivité et l'environnement, Université de Lille, CNRS UMR 8516, F-59000 Lille, France*

[g.] *CNRS, ENS de Lyon, LCH – Laboratoire de Chimie, UMR 5182, 69342, Lyon cedex 07, France*

[h.] *Institut Universitaire de France, 5 rue Descartes, F-75005 Paris, France*

[**]Corresponding author: mohamed-nawfal.ghazzal@universite-paris-saclay.fr

Experimental Section

Materials

Tetraethyl orthosilicate (TEOS, Sigma-Aldrich, 98%), titanium (IV) isopropoxide (TTiP, Sigma-Aldrich, 97%), Gold(III) chloride trihydrate ($HAuCl_4 \cdot 3H_2O$, ≥99.9% Sigma-Aldrich), Chloroplatinic acid hydrate ($H_2PtCl_6 \cdot xH_2O$, ≥99.995%, Sigma-Aldrich), Silver nitrate ($AgNO_3$, ≥99.0%, Sigma-Aldrich), Potassium tetrachloropalladate(II) (K_2PdCl_4, ≥99.99%, Sigma-Aldrich) terakis (hydroxylmethyl) phosphonium chloride (THPC, Sigma-Aldrich, 80% in H_2O), 3-aminopropyl-trimethoxy silane (APTMS, Alfa Aesar, 97%), ammonium hydroxide 28% (NH_4OH, Sigma-Aldrich), absolute ethanol (EtOH, VWR Chemicals), methanol (MeOH, Honeywell Fluka), Potassium sulfate (K_2SO_4, ≥99.0%, Sigma-Aldrich), Acetonitrile (CH_3CN, ≥99.9%, Sigma-Aldrich), Phosphoric acid (H_3PO_4, ≥85% in H_2O, Honeywell-Fluka), 2,4-Dinitrophenylhydrazine (DNPH, $(O_2N)_2C_6H_3NHNH_2$, ≥99.0%, Fluka), Argon gas (Ar, ≥99.9999%, Alphagaz 2 – Air Liquide), Mill-Q water. All chemical reagents were of analytical grade and using without further purification.

Synthesis of core@shell photocatalysts

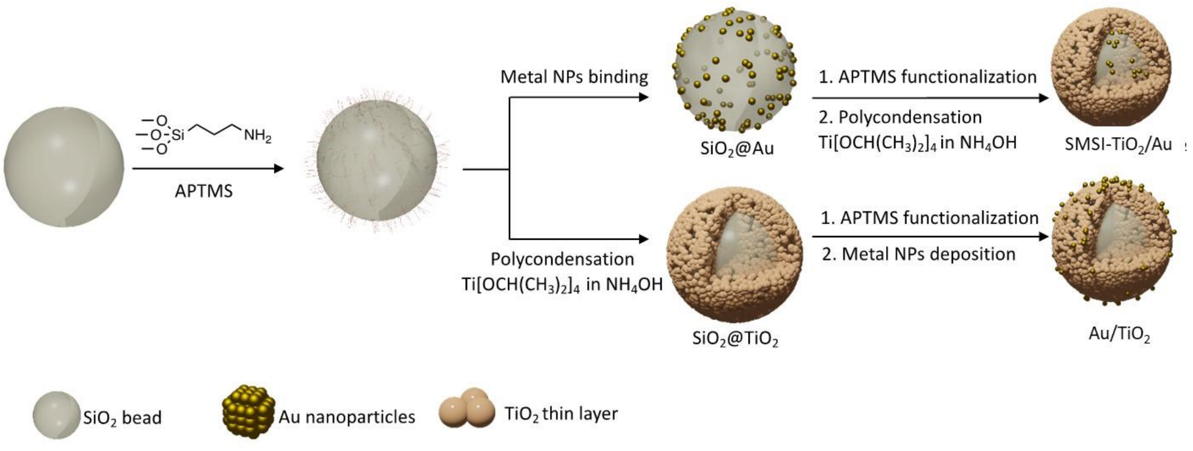

Figure S1| Schematic illustration of synthesis core@shell nanostructures: SMSI-like configuration and conventional configuration.

Synthesis of monodispersed SiO_2 microspheres.

Silica beads with the expected size of ~200 nm were synthesized using the Stöber method.[1] A mixture in a borosilicate bottle containing 42 mL of EtOH and 35 mL of distilled water was stirred for 10 minutes at room temperature before adding 15 mL of NH_4OH 28% to adjust pH.

Once the temperature had been raised to 40°C, 4 mL of TEOS was pipetted to the bottle, and the solution was left to stir for 1h. After that, the white precipitate was recovered by centrifugation, washed 3 times with distilled water, and dried at 70°C overnight.

APTMS-functionalization of substrates

To deposit metal nanoparticles onto the SiO_2 surface, we employed APTMS as a coating agent, which possesses distinct terminal groups: one amine group (-NH_2) and three methoxysilane groups (Si-OCH_3).[2] While the amine group exhibits a strong affinity toward noble metals and transition metal oxides, the methoxysilane groups enable APTMS to anchor to the SiO_2 surface through hydroxyl groups (OH). The surface of SiO_2 microspheres needed activation to enhance hydrophilicity and increase the quantity of hydroxyl groups. Specifically, we dispersed 300 mg of the raw SiO_2 nanoparticles in 10 mL mixture of CH_3OH and HCl (37%) (1:1 v/v ratio) and sonicated them for 30 minutes before thoroughly washing three times with 10 mL EtOH each. The activated SiO_2 particles were then redispersed in 10 mL of EtOH by ultrasonication. Next, 0.1 mL of APTMS was added to the mixture under stirring. After six hours of stirring, the APTMS-functionalized SiO_2 was centrifuged and washed three times with 10 mL EtOH each. The functionalization process was repeated for SiO_2@M and SiO_2@TiO_2 before coating with a TiO_2 shell and metallic nanoparticles.

Synthesis of Gold, Palladium, and Silver nanoparticles

Au, Pd, and Ag nanoparticles were synthesized using the Duff and Baiker method.[3] First, 1.5 mL of NaOH (0.2 M) was added to 45 mL of distilled water under vigorous stirring. After 5 minutes, 1 mL of THPC 0.067 M was pipetted into the basic mixture, followed by an adequate amount of corresponding precursor: $HAuCl_4.3H_2O$ (7.847 mg), K_2PdCl_4 (12.08 mg) , and $AgNO_3$ (6.18 mg), to reach a mass ratio of 1 wt% with respect to TiO_2. The previous colourless solution change to brown colour immediately, indicating the formation of Au^0. The solution was stirred around 30 minutes before being dark at room temperature. The final color of the gold nanoparticle solution is similar to red wine in four days.

Synthesis of Platinum nanoparticles

We also used Duff & Baiker method to synthesize Pt nanoparticles, and small modification of the procedure was employed a little modification. Firstly, 45 mL of water was heated to 90°C in a borosilicate bottle. Next, the pH was adjusted by adding 0.5 mL of NaOH (1.2 M) before adding 1 mL of THPC (0.057M). After stirring for 1 minute, 8.245 mg of H_2PtCl_6 was quickly pipetted into the bottle. The color of the solution turned immediately to

light black. The reaction finished after stirring for 15 minutes later.

Deposition of (Au/Ag/Pd/Pt) on substrate's surface

In this procedure, the surficial APTMS-functionalized substrate, either SiO_2 or SiO_2@TiO_2, was dispersed into solutions containing the desired metal nanoparticles. 0.1 mL of NaOH 0.2M was added to adjusted pH into basic media. The mixture was stirred for at least 4 hours until achieving uniformly coloured materials, ensuring homogeneity throughout the solution. The materials were washed 3 times with 10 mL water each and dried overnight.

Coating cores with TiO_2 thin shell

SiO_2@M and SiO_2 cores were coated with TiO_2 shell through hydrolysing TTiP in basic media. In detail, the core nanoparticles were first dispersed in 20 mL of EtOH and 0.5 mL of H_2O. Next, the pH was adjusted at 12.4 by adding 0.3 mL of NH_4OH (28%), followed by stirring for 20 minutes. Concurrently, 1.5 mL of TTiP was dissolved in 50 mL of absolute EtOH (99%). This solution was slowly added to the basic solution containing core nanoparticles while stirring at room temperature. After aging for two hours, the final core-shell nanocomposites were obtained by centrifugation, followed by careful washing three times with 10 mL absolute EtOH each, and finally drying at 70°C overnight. The photocatalysts must be calcined at 500°C for 2 hours with a temperature elevation speed of 2°C/minute.

Table S1| Synthesis parameters for SMSI-like core@shell nanostructures

Samples	m_{SiO2} (mg)	Precursor metal NPs (mg)		TTiP (mL)	m_{metal}/m_{TiO2}
SMSI-TiO_2/Au	300	$HAuCl_4.3H_2O$	7.847	1.5	1%
SiO_2-TiO_2/Pt	300	H_2PtCl_6	8.245	1.5	1%
SiO_2-TiO_2/Pd	300	K_2PdCl_4	12.08	1.5	1%
SiO_2-TiO_2/Ag	300	$AgNO_3$	6.18	1.5	1%

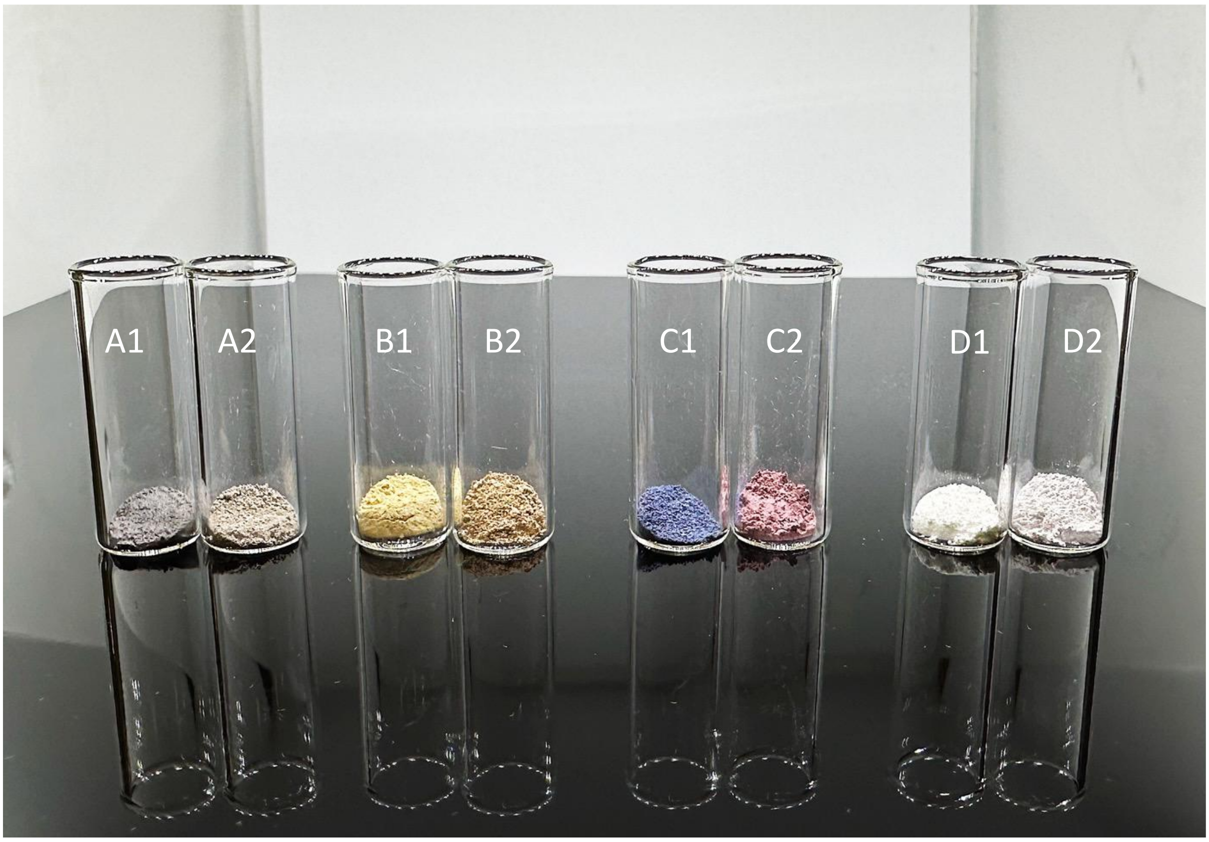

Figure S3| Images of core@shell photocatalysts: **(A1)** SMSI-TiO_2/Pt, **(B1)** SMSI-TiO_2/Pd, **(C1)** SMSI-TiO_2/Au, **(D1)** SMSI-TiO_2/Ag, **(A2)** Pt/TiO_2, **(B2)** Pd/TiO_2, **(C2)** Au/TiO_2, **(D2)** Ag/TiO_2.

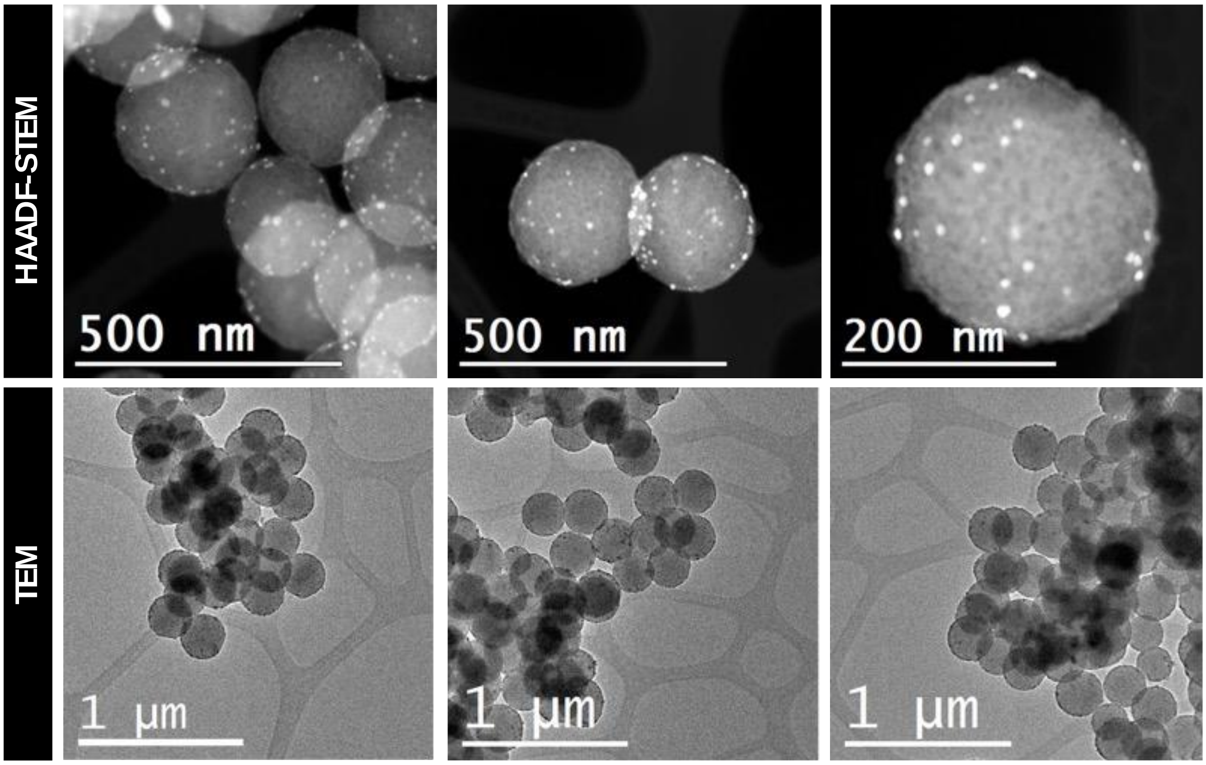

Figure S2 | Overview images of gold-embeded core@shell nanostructure (SMSI-TiO_2/Au) captured using TEM and HAADF-STEM techniques.

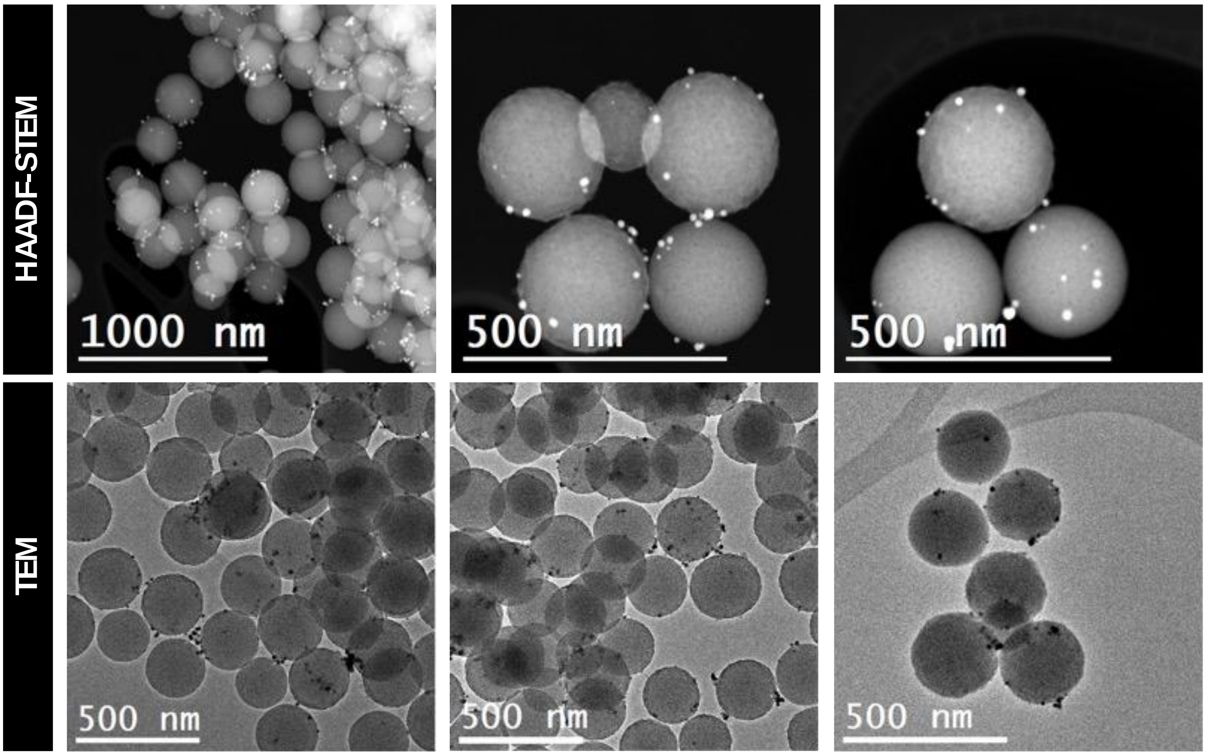

Figure S4| Overview images of gold-deposited $SiO_2@TiO_2$ nanostructure (Au/TiO_2) captured using TEM and HAADF-STEM techniques.

In the conventional configuration, deposited Au NPs (diameter approx. 10 nm, **Figure S5C** and **Figure S5D**) had a cubic FCC structure, but along its [111] zone axis instead; none of TiO_2 overlayer covered up the Au NPs, evidenced by the absence of TiO_2 in the electron diffraction pattern (**Figure S5D**). The scanning transmission electron microscopy coupled with electron energy loss spectroscopy (STEM-EELS) provided further morphological observation and chemical mapping of different elements (Ti, O, Si, and Au), hence visualizing their distribution within the nanostructures (**Figure S5F**). The chemical mapping indicated the core of the Au/TiO_2, mainly consisting of Si and O. Au NPs were deposited on the surface of TiO_2 shell (**Figure S5F**) in Au/TiO_2. The EELS (**Figure S5G**) confirmed that Au NPs were not encapsulated. The electron energy loss spectroscopy (EELS) intensity performed on selected

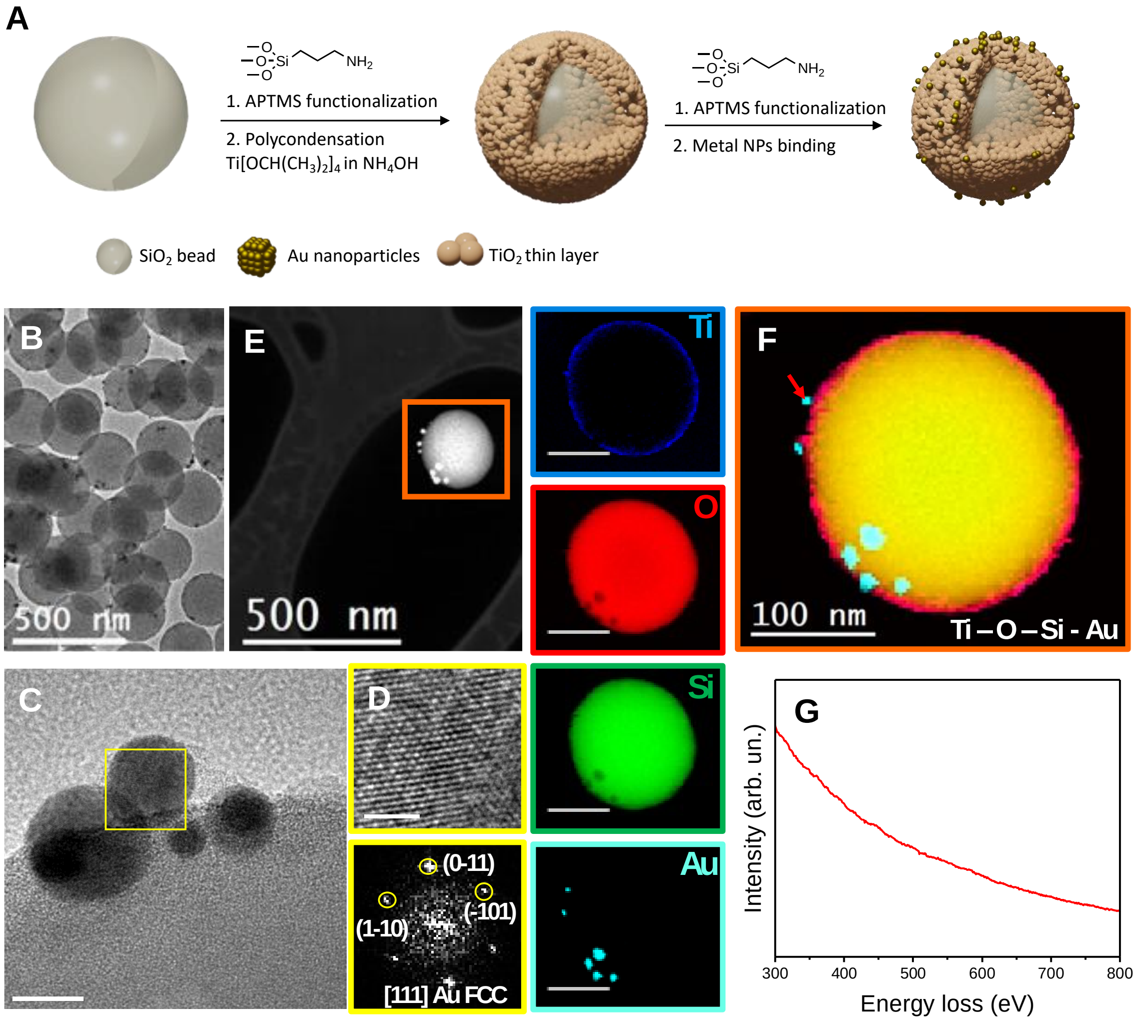

Figure S5| Microscopic characterizations of gold-deposited core@shell photocatalyst. (A) Soft-chemistry synthesis pathway for Au/TiO_2. **(B)** Localized bright-field TEM image of Au/TiO_2. **(C)** HRTEM image of Au/TiO_2. **(D)** HRTEM of a localized Au nanoparticle and electron diffraction pattern. **(E)** STEM-HAADF image of an Au coted SiO_2@TiO_2 core-shell. **(F)** STEM-EELS mapping of separated elements and their combination: Ti L edge at 456 eV (ultramarine blue), O K edge at 532 eV (scarlet red), Si K edge at 1839 eV (kelly green) and Au M edge at 2206 eV (turquoise blue). **(G)** EELS spectrum at a deposited Au NP (red arrow in **(F)**).

area containing Au NPs (red arrow, **Figure S5F**) further demonstrated the formation of the TiO_2 overlayer on the top of the Au NPs, assigned to Ti L edge at 456 eV and O K edge at 532

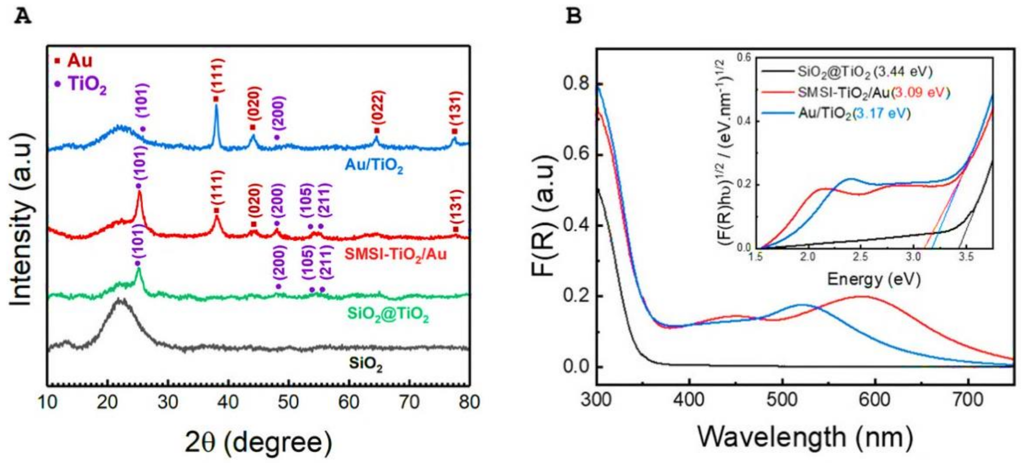

Figure S6| (A) Powder XRD diffractogram with variable samples. **(B)** Diffuse reflectance spectra in the range from 300 nm to 700 nm (inset: Tauc plots).

eV in the EELS spectrum is absent in Au/TiO_2 (**Figure S5G**).

The crystal structure of our photocatalysts was further characterized by powder X-ray diffraction (XRD). The diffractograms in **Figure S6A** displayed wide peaks at 22°, indicative of an amorphous SiO_2 core. Moreover, distinctive peaks at 25.3° were observed in the diffractograms of SiO_2@TiO_2 and SMSI-TiO_2/Au, identifying the lattice plane (101) of the TiO_2 anatase phase, while the intensity of this peak diminished in the diffractogram of Au/TiO_2. Additional characteristic peaks of TiO_2 anatase were evident in the diffractogram, indicating the presence of their respective lattice planes, including (200), (105), and (204). Characteristic peaks assigned to lattice planes (111), (020), (022), and (131) of Au FCC were recorded in diffractograms of both SMSI-like and conventional samples. This is in agreement with the previous SAED results. The intensity of these peaks was more significant for deposited Au. We reckon that the diffraction of the metallic Au phase dominated the TiO_2 phase, leading to the decreasing intensity of TiO_2 anatase peaks.

Au-mediated samples absorbed both UV and visible light, while the absorbance of Au-free sample (SiO_2@TiO_2) was limited to UV range. Au/TiO_2 exhibited a broadband absorption cantered at 520 nm, characteristic of the local surface plasmon resonance (LSPR) of gold. The band was shifted by around 70 nm in the case of SMSI-TiO_2/Au. The red shift is due to the increase in the refractive index of the surrounding dielectric environment when Au nanoparticles were encapsulated by TiO_2 overlayer.[4] Indeed, in the Au/TiO_2 system, AuNPs are partially exposed to air (n_air = 1) and partially in contact with TiO_2 ($n_{TiO2} \approx 2.1$)[5]. In contrast, in the strong metal–support interaction (SMSI) system, SMSI-TiO_2/Au, the Au NPs are encapsulated with TiO_2 and in contact with SiO_2 ($n_{SiO2} \approx 1.4$)[6]. The redshift in the maximum LSPR wavelength is due to the change in refractive index between air and TiO_2.

The band gap energy of each sample was estimated from Tauc plot method[7] (inset **Figure S6B**). The bandgap energy is estimated to be 3.44 eV, 3.17 eV, and 3.09 eV for SiO_2@TiO_2, Au/TiO_2, and SMSI-TiO_2/Au, respectively.

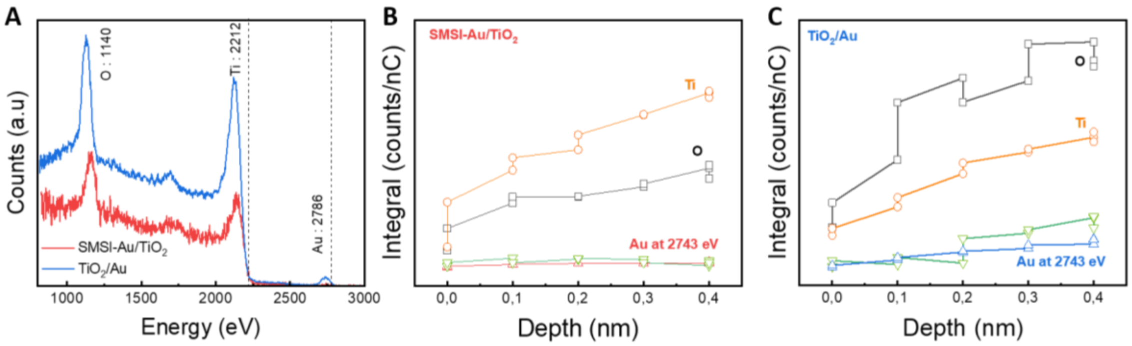

Figure S7| Initially, the signals recorded at very low ion doses were moderate, indicating the removal of contaminants (carbon, hydrogen, etc.) by the ion beam; these contaminants were disregarded for analysis. Due to the absence of standardized measurements for nanoparticles dispersed on a porous support and the initial presence of contamination layers, the translation of results to a probed depth expressed in nanometres was deemed unfeasible. LEIS signals were recorded after 20 seconds of sputtering by He^+ ions. **(A)** Low energy ion scattering spectra of SMSI-TiO_2/Au and Au/TiO_2. **(B)** LEIS depth profile of SMSI-TiO_2/Au. **(C)** LEIS depth profile of Au/TiO_2.

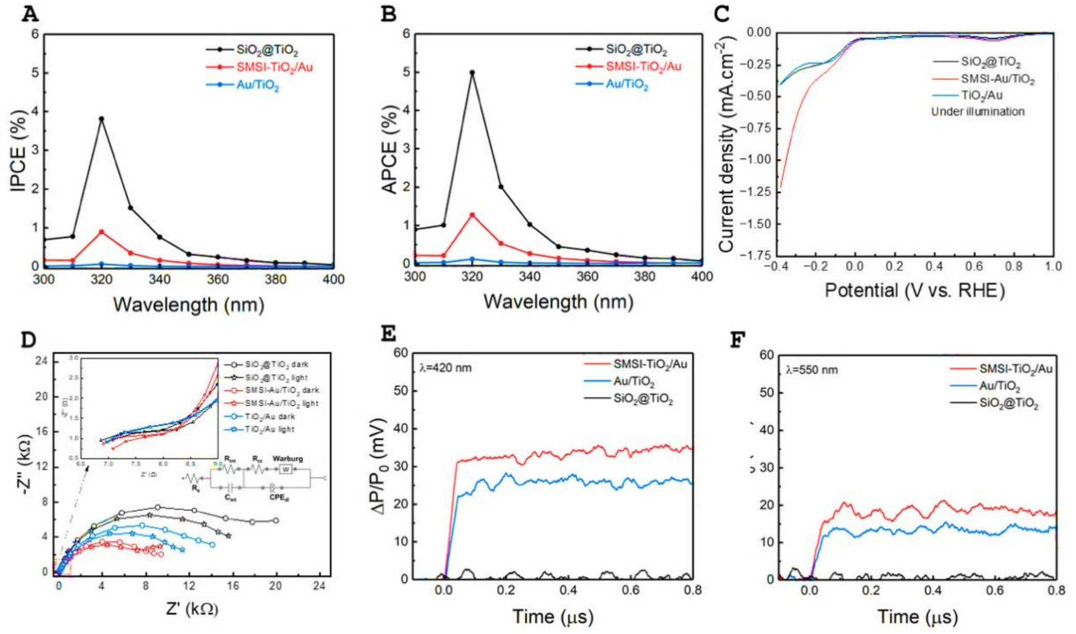

Figure S8| Photoelectrochemical characterizations and charge carrier dynamics: (A) Incident photon-to-current efficiency (IPCE) spectra. **(B)** Absorbed photon-to-current efficiency (APCE) spectra. Photoelectrochemical measurements performed at a fixed bias voltage 0.6 V vs. Ag/AgCl (sat. KCl) in Na_2SO_3 0.1 M buffered at pH = 7 under intermittent AM 1.5G solar irradiation. **(C)** Linear sweep voltammetry (LSV) plots of the photoelectrodes with cathodic sweeping in K_2SO_4 0.5M buffered at pH=7, scan rate 50mV.s^{-1} under AM 1.5G beam light. **(D)** Electrochemical Impedance Spectroscopy (EIS) Nyquist plots of the front-contact photoelectrodes recorded in dark and AM 1.5G illumination in K_2SO_4 0.5M. Time-resolved microwave conductivity (TRMC) signals of the samples triggered by visible laser irradiation **(E)** $I_{ex} = 7.072\ mJ.cm^{-2}$ at $\lambda = 420\ nm$, and **(F)** $I_{ex} = 6.562\ mJ.cm^{-2}$ at $\lambda = 550\ nm$.

Table S3| EIS fitting parametres of different TiO_2-based nanostructures.

Samples		R_s (Ω)	R_{int} (Ω)	R_{ct} (Ω)	C_{int} (μF)	CPE_{dl} (μF)	n
$SiO_2@TiO_2$	light	6.36	1.84	15878	1.76	24.17	0.84
	dark	6.54	1.84	18633	1.64	22.51	0.85
Au/TiO_2	light	6.43	1.78	11201	1.78	20.56	0.81
	dark	6.51	1.81	13262	1.87	20.69	0.83
SMSI-TiO_2/Au	light	6.67	1.47	7304	2.33	26.02	0.85
	dark	6.27	1.61	8711	1.49	25.32	0.82

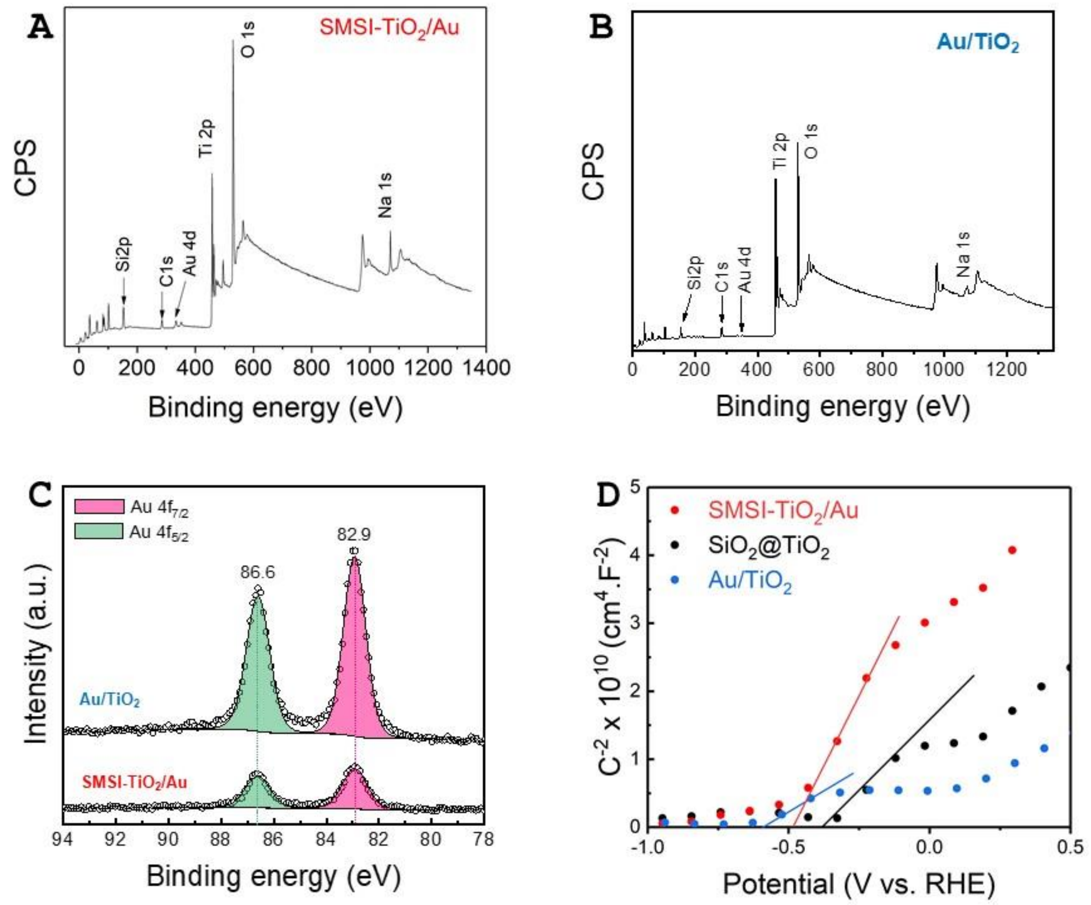

Figure S9| Full XPS spectra of elements: **(A)** SMSI-TiO_2/Au. **(B)** Au/TiO_2. **(C)** X-ray photoelectron spectroscopy (XPS) spectra of Au 4f. **(D)** Mott-Schottky plots in K_2SO4 0.5M buffered pH=7 at frequency of 1 kHz and AC amplitude of 5 mV.

Computational Details

Computations were performed on a 5x2x1 unit cell of anatase TiO_2(101) with cell parameters a = 18.98, b = 20.33 and c = 30.0Å for plain titania, Au1/TiO_2 and Au55/TiO_2; and a 4x1x1 unit cell used for Au(111)/TiO_2 and SMSI-TiO_2/Au(111) systems. The titania slab thickness was approximately 1 nm for all systems, comparable to experiments. The electronic structure was evaluated at the gamma point using CP2K combining GTH pseudopotentials, a DZVP MOLOPT basis set, and Fermi Dirac Smearing of 300K for metallic systems. The electronic convergence was set at 5.10^{-6}H. We used a PBE functional supplemented with a D3 correction to take into account dispersion. A Hubbard correction was introduced to evaluate vacancies formation with a U value of 4 eV and using a spin-polarized evaluation of the wavefunction. We had to use a U value of 2eV for the Ag case and hence used a U=2eV for all metals when comparing them in **Figure 3H**. Geometry optimization were performed using the BFGS optimizer and default convergence parameters of CP2K. Optimized structures are provided as supplementary materials.

The electronic structural analysis of the different surfaces was performed by projected density of states (PDOS) analysis, which appears to be small mid-gap peaks for surfaces with oxygen vacancies. For bulk vacancies, these are close to the conduction band edge, but for surface vacancies, they shift closer towards the valence band edge. The presence of Au amplifies this shift even further. For anatase surfaces completely covered with Au, the impurity peaks disappear from the mid-gap region when the oxygen vacancy is at the gold-titania interface, possibly getting buried in the valence band region. The bulk and bare surface O_{vac} sites continue to show the mid-gap states, but they are located between the valence band and the Fermi level.

Adsorption energies are computed as the difference between the adsorbed structure ($E_{M@slab}$)and the pristine slab (E_{slab})and the corresponding molecule isolated in a large cubic box (E_M). A stabilizing adsorption yields to a negative adsorption energy:

$$E_{ads} = E_{M@slab} - E_{slab} - E_M$$

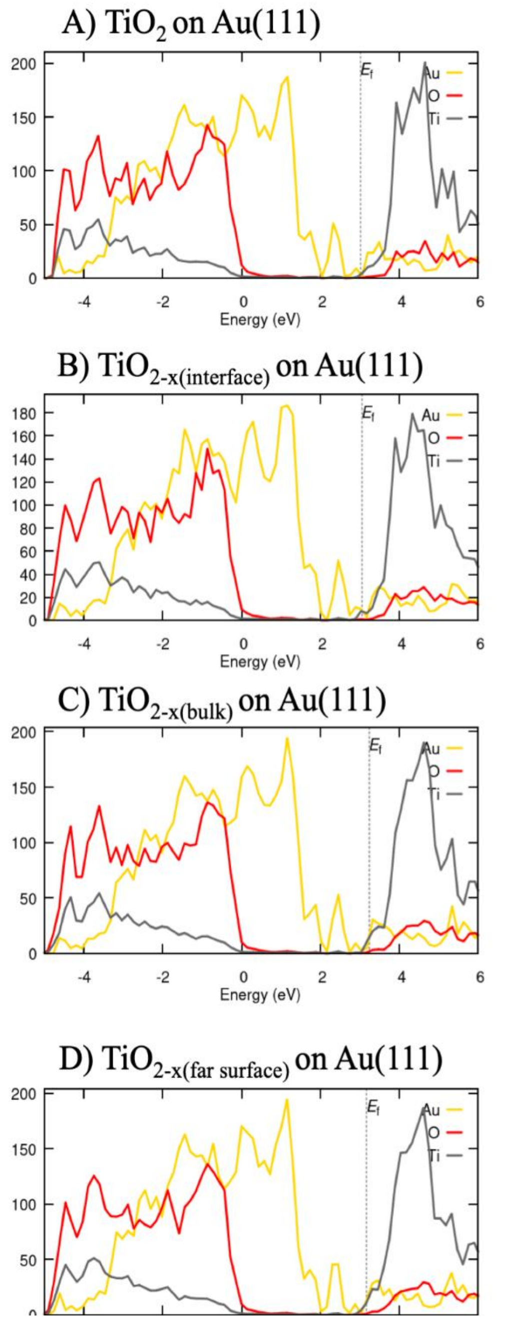

Figure S10| PDOS for SMSI-TiO_2/Au (a) without and with oxygen vacancies at (b) Au-TiO_2 interface, (c) TiO_2 bulk and (d) TiO_2 surface far from Au layer

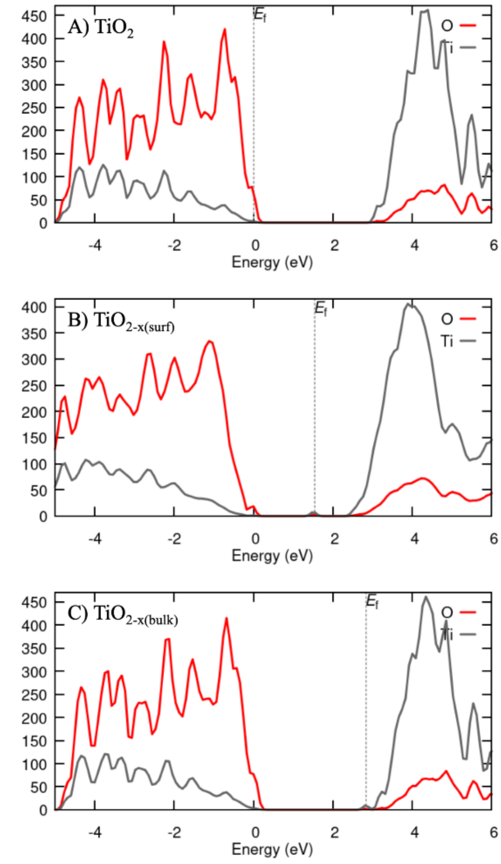

Figure S11| PDOS for anatase TiO_2 with and without oxygen vacancies

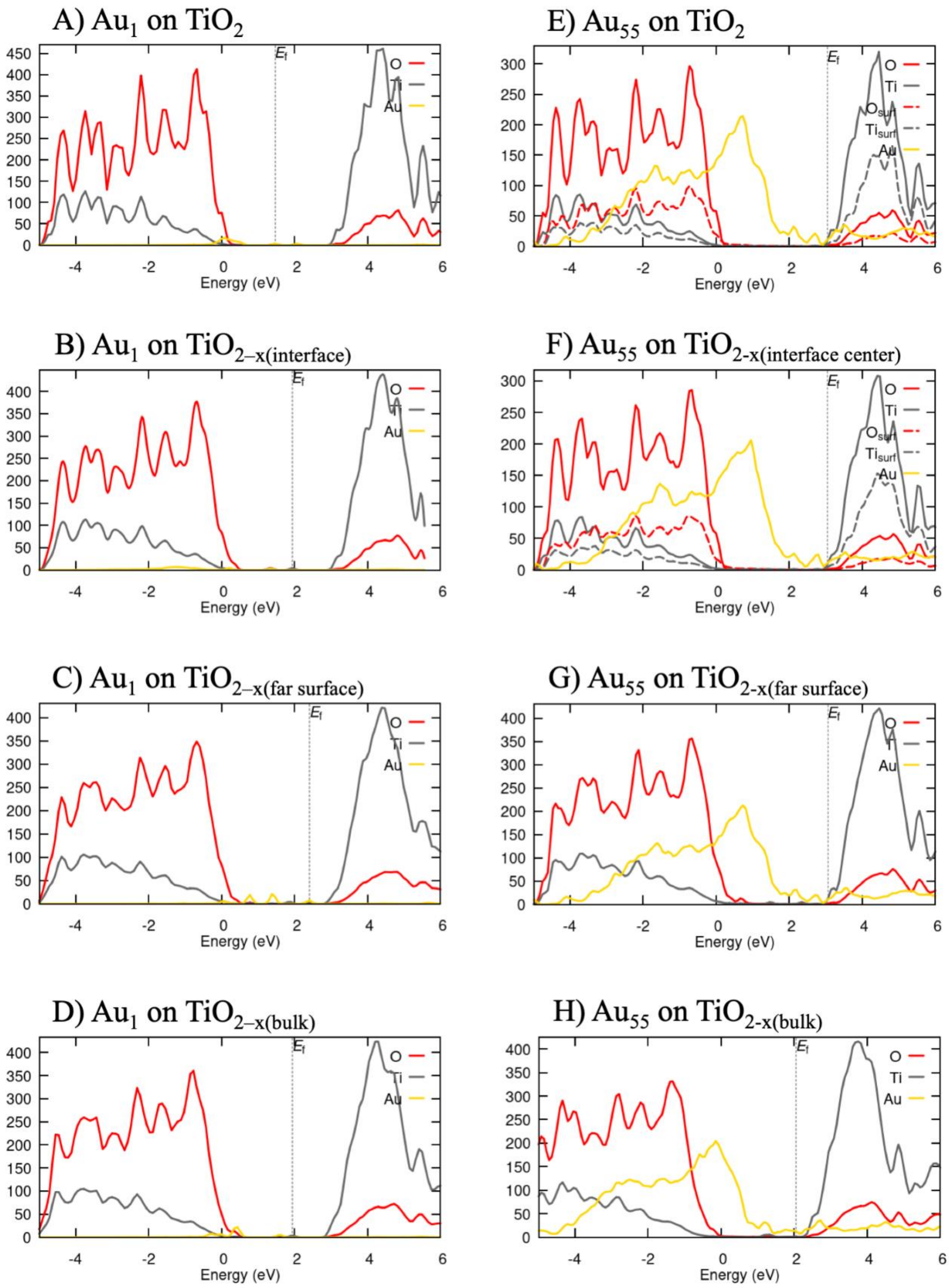

Figure S12| PDOS for single Au atom (A-D) and Au55 cluster(E-H) on anatase TiO_2

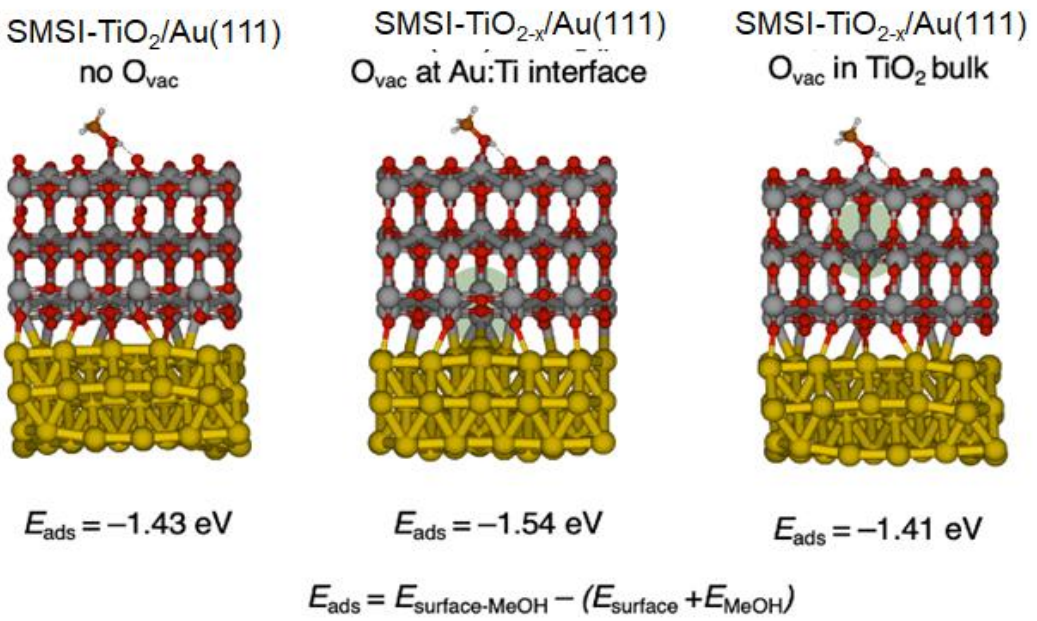

Figure S13| Adsorption energies for methanol on SMSI-Au/TiO_2. Green highlighted region shows the location of oxygen vacancies.

Table S4| Molecular adsorption energies in eV for water and methanol on Au:TiO_2 surfaces without or with different oxygen vacancy (O_V) sites

Catalyst model			Adsorption Energy	
Structure	Model of	O_V site	H_2O	MeOH
TiO_2	SiO_2@TiO_2	None	−0.91	−1.06
SMSI-TiO_2/Au(111)	SMSI-TiO_2/Au	None	−1.27	−1.43
Au(111)/TiO_2	Au/TiO_2	None	−0.51	−0.35
SMSI-TiO_{2-x}/Au(111)	SMSI-TiO_2/Au	Au:Ti interface	−1.45	−1.54
		Bulk	−1.29	−1.41
Au(111)/TiO_{2-x}	Au/TiO_2	Au:Ti interface	−0.59	−0.60
		Bulk	−0.28	−0.30

A

Element		Pd	Pt	Au	Ag
Atomic number		46	78	79	47
Group number		10	10	11	11
Electronegativity		2.2	2.28	2.54	1.93
Work Function (eV)		5.22-5.6	5.1-5.9	5.1-5.47	4.2-4.7
Total charge on metal	No Ov	-0.162	-0.722	-0.129	1.068
	O_v,s	-0.643	-0.961	-0.189	0.973
	O_v,i	-0.733	-1.197	-0.658	0.867
E_O_v	O_v,s	2.65	2.85	5.04	5.25
	O_v,i	2.51	2.53	3.70	4.45

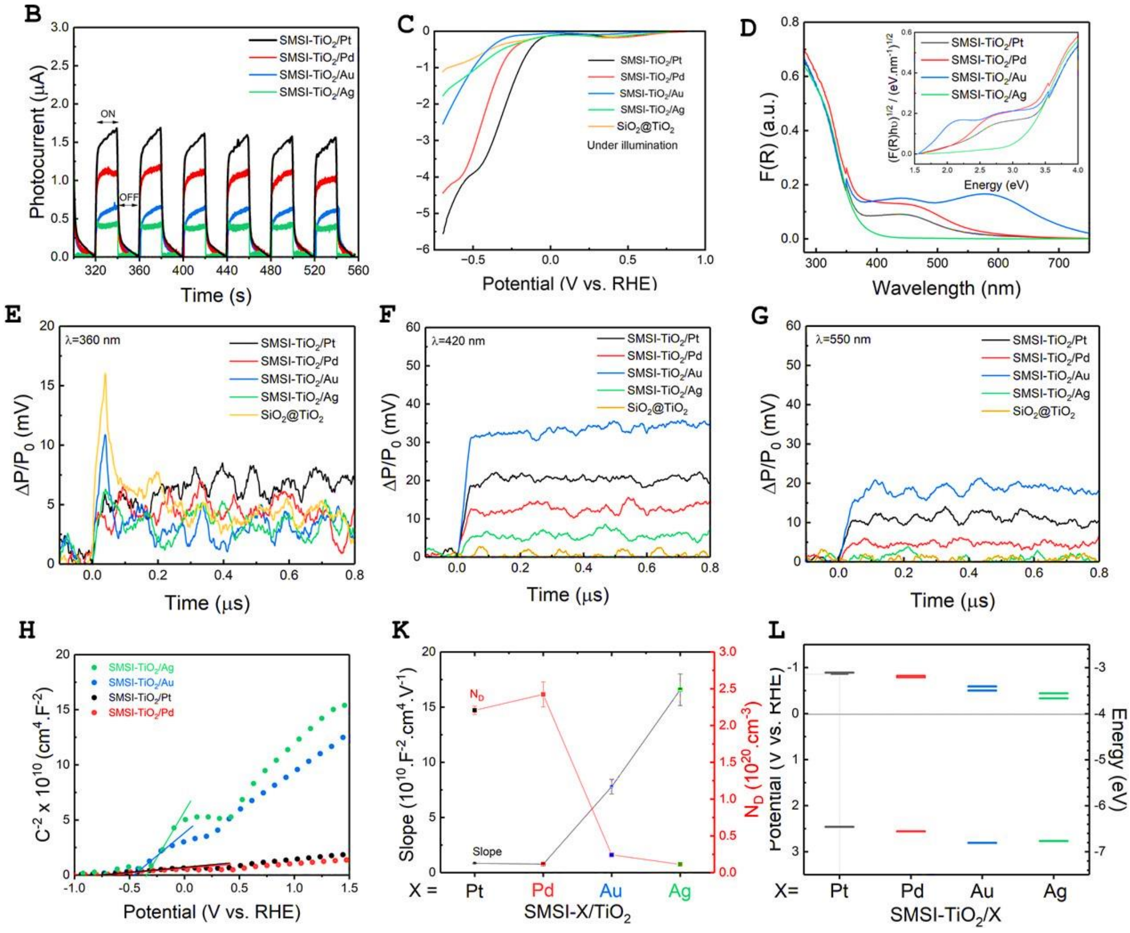

Figure S14| (A) Oxygen vacancy formation energy and metal charge within SMSI-like nanostructures with Pd, Pt, Au, and Ag. **(B)** Amperometry I-t curves of the photoelectrodes at a fixed bias voltage 0.6 V vs. Ag/AgCl (saturated KCl) under intermittent AM 1.5G solar irradiation in K_2SO_4 0.5M buffered pH = 7. **(C)** Linear sweep voltammetry (LSV) plots of the photoelectrodes with cathodic sweeping in K_2SO_4 0.5M buffered at pH=7, scan rate 50mV.s^{-1} under AM 1.5G beam light. **(D)** UV-Vis Diffuse Reflectance Spectra (DRS). Time-resolved Microwave Conductivity (TRMC) signals of the samples triggered by laser irradiation: **(E)** $I_{ex} = 1.396\ mJ.cm^{-2}$ at $\lambda = 360\ nm$, **(F)** $I_{ex} = 7.072\ mJ.cm^{-2}$ at $\lambda = 420\ nm$, and **(G)** $I_{ex} = 6.562\ mJ.cm^{-2}$ at $\lambda = 550\ nm$. **(H)** Mott-Schottky plots in K_2SO4 0.5M buffered pH=7 at frequency of 1 kHz and AC amplitude of 5 mV. **(K)** Donor

(oxygen vacancy) density, N_D, of the photocatalysts and its inverse proportion to the slope of Mott-Schottky plots. **(L)** Band alignments of SMSI-like nanostructures containing different metal nanoparticles.

Figure S14.E shows the TRMC signal triggered by 360 nm laser excitation, activating only TiO_2. Coupling metal species with TiO_2 reduces signal intensity as metals collect electrons. In the 0–0.1 µs range, SMSI-TiO_2/Au and SMSI-TiO_2/Ag exhibit a peak followed by a sharp decay, indicating short charge carrier lifetimes. The signals of SMSI-TiO_2/Pt and SMSI-TiO_2/Pd do not show such a sharp increase. The results suggested that there is a fast electron transfer from TiO_2 to Pt/Pd during the laser pulse (<8ns), followed by a slow release of the electron into TiO_2, explaining the plateau for Pd and Pt metals.

Characterization techniques

TEM, HRTEM, and STEM images were captured by a field emission gun FEI Tecnai F20 microscope. HRTEM was acquired with a condenser aperture of 100 µm, no objective aperture, spot size 3, and a BM-UltraScan CCD camera. STEM-EELS) chemical mapping was acquired with a condenser aperture of 70 µm, no objective aperture, nominal camera length of 30mm, spot size 6, and Gatan EF-CCD camera. STEM-EELS was acquired with a condenser aperture of 70 µm, no objective aperture, nominal camera length of 30mm, spot size 6, and Gatan EF-CCD camera. An annular dark field detector (DF4) was combined to output STEM-HAADF images.

Powder X-ray diffractograms were recorded on an Aeris powder X-ray diffraction (XRD) diffractometer from Malvern Panalytical Corporation. The measurement was operated at 40 kV and 15 mA with a monochromatized Cu Kα radiation (λ = 1.5406 Å) and a linear VANTEC detector. Raw data were analyzed using X'Pert high score plus software.

X-ray photoelectron spectroscopy (XPS) was performed on a Kα spectrometer from ThermoFisher, equipped with a monochromate X-ray Source (Al Kα, 1486.7 eV) with a spot size of 400 µm. The hemispherical analyzer was operated in CAE (Constant Analyzer Energy) mode, with a pass energy of 200 eV and a step of 1 eV for the acquisition of surveys spectra, and a pass energy of 50 eV and 20eV and a step of 0.1 eV for the acquisition of narrow spectra. A "dual beam" flood gun neutralized the charge build-up. Spectra were treated using CasaXPS software. A Shirley-type background subtraction was used, and peak areas were normalized using the Scofield sensitivity factors. The peaks were fitted with mixed Gaussian-Lorentzian line shapes (70% of Gaussian character). The binding energies were calibrated against the Ti

$2p_{3/2}$ binding energy set at 458.5 eV, as the carbon signal is weak.

Low-energy ion scattering (LEIS) spectroscopy was performed on a Qtac100 spectrometer (IONTOF GmbH) at the Imperial College of London, the United Kingdom. A 3 keV He^+ beam at normal incidence was selected for qualitative surface analysis. The ionic dose per spectrum was first limited to 1 x 10^{15} ions.cm^{-2} for the core-shell samples to complete the surface analysis and the composition of the sub-surface. The data were processed using the SurfaceLab software from the same manufacturer. LEIS signals were decomposed using the experimental lineshapes of reference materials (at 2379 eV for Cu and 2212 eV for Ti) to take the isotopic distribution of each metal into account.

ICP-OES (Inductively Coupled Plasma Optical Emission Spectrometry) analysis was performed using Agilent 720-ES ICP-OES equipment combined with Vulcan 42S automated digestion system. Vulcan 42S is an automated digestion system combining the two essential steps in sample preparation prior to analysis by ICP: sample digestion followed by sample work-up. The digestion procedure was as follows: firstly, 10 mg samples were weighted and 2.4 mL of aqua regia and 1 mL of HF were added to each sample by the robot then heated for 2 hours up to 110°C (this step is repeated 3 times), followed by 1 hour heating up to 110°C. Almost all the digester components are made of Teflon to avoid corrosion with the use of acids. Questrom uses a highly efficient fume removal after neutralizing the acid vapour thereby avoiding cross-contamination.

The optical properties of our photocatalysts were investigated using steady-state UV-visible diffuse reflectance spectroscopy (UV-vis DRS) ranging from 200 nm to 800 nm. The technique was performed on a Cary 5000 UV-Vis-NIR spectrophotometer; therein, $BaSO_4$ referred to zero baseline correction. The band gap energy values of samples were estimated via the Tauc equation proposed by Tauc *et al.*[7]:

$$(\alpha h\nu)^{\frac{1}{\gamma}} = B(h\nu - E_g)$$

where α is the extinction coefficient, h is Planck's constant, ν is the photons' frequency, B is constant, and E_g is the bandgap energy. The factor γ represents the nature of electron transition in semiconductors; γ = 2 for indirect transition and γ = 0.5 for direct transition. TiO_2 is a semiconductor performing indirect transition[8], leading to γ = 2. To cancel the influence of light scattering on estimating the bandgap energy, we used of Kubelka-Munk function[9]:

$$F(R) = \frac{(1-R)^2}{2R}$$

where R is the reflectance used to deduce absorption spectra. Replacing F(R) on the Tauc equation provides an equation:

$$(F(R)h\nu)^{\frac{1}{\gamma}} = B(h\nu - E_g)$$

Time-resolved microwave conductivity (TRMC) allows the study of the lifetime and dynamics of charge carriers that are generated at or transferred to the surface of TiO_2 via the change in conductivity (σ) of the sample induced by a pulsed laser. In particular, mobilized charge carriers (e^-/h^+) induce a relative change in the reflected microwave power ($\Delta P(t)/P$). The following expression gives the relation between conductivity and microwave absorption:

$$\Delta P(t)/P \; = \; A\, \Delta\sigma(t) \; = \; A\; e\; \Sigma i\; \Delta ni(t)\; \mu i$$

in which Δni is the free charge carrier density, μi is the free charge carrier mobility, and A is the sensitivity factor. A TRMC signal would identify two parameters: maximum conductivity value I_{max} and time decay $I(t)$. The maximum intensity determines the concentration of free charge carrier density created during irradiation. At the same time, the decay $I(t)$ corresponds to the trapping, charge carrier recombination, or electron/proton transfer in time t (from the nanosecond to the microsecond timescale). TRMC setup includes a laser source and a microwave source. The laser source is OPO laser (EKSPLA, NT342B), characterized by the emission of wavelengths from 225 to 2000 nm, with a half-height pulse width of 8 ns and a pulse repetition rate of 10 Hz. Continuously generated microwaves of 30 GHz frequency are derived from a Gunn GKa-300 diode.

(Photo)electrochemical testing involving transient photocurrent amperometry (TPC), cyclic voltammetry (CV), and electrochemical impedance spectroscopy (EIS) was carried out in a three-electrode electrochemical cell with/without illuminating by AM 1.5G solar simulator. Working electrodes were fabricated by dropping ultra-dispersed solution ($1 mg.mL^{-1}$) of our samples on fluorine-doped tin oxide-coated glass slides of 2.5 cm-2 (FTO glass) before drying overnight at room temperature. We adopted a platinum disk counter electrode and an Ag/AgCl (3M KCl) reference electrode ($E_{R.H.E} = E_{Ag/AgCl} + 0.210 + 0.059 \times pH$). Experimental parameters and processes were controlled by potentiostat PGSTAT101 Metrohm Autolab. Incident photon-to-current efficiency (IPCE) and absorbed photon-to-current efficiency

(APCE) were measured using light from a 1000 W Xe Arc lamp passing through Czerny-Turner monochromators (Quantum Design, MSH-300). The sequence at each wavelength was 10 s of dark and 10 s of illumination, and the current was collected at 10 points/second. The photoelectrochemical setup remains identical and biases at 0.6 V vs. Ag/AgCl (1.23 V vs. RHE). IPCE and APCE were calculated using the following equations:

$$IPCE(\lambda) = \frac{electrons/cm^2/s}{photons/cm^2/s} = \frac{|j_{ph}(mA/cm^2)| \times 1239.8(V \times nm)}{P_{mono}(mW/cm^2) \times \lambda(nm)}$$

$$APCE\ (\lambda) = \frac{IPCE(\lambda)}{1 - 10^{-A}}$$

in which j_{ph} is photocurrent, P_{mono} is the monochromated illumination power intensity, λ is the wavelength at which this illumination power is measured, A is absorbance from Beer-Lambert law

The Mott-Schottky relationship is described in the following equation:

$$\frac{1}{C_{SC}^2} = \frac{2}{e\varepsilon_0\varepsilon_r N_D}(\phi - \phi_{fb} - \frac{kT}{e})$$

where C_{SC} is capacitance density (F.cm^{-2}), e is an elementary charge (C), ε_0 is permittivity in vacuum (F.cm^{-1}), ε_r is the dielectric constant of TiO_2 (75),[10] k is Boltzamann's constant (J.K^{-1}), T is the temperature (298 K). The donor density, N_D (oxygen vacancy density), can be obtained from the slope of Mott-Schottky fitting as:

$$Slope = \frac{2}{e\varepsilon_0\varepsilon_r N_D}(F^{-2}.cm^4.V^{-1})$$

Photocatalytic assessments

Photocatalytic H_2 evolution reactions

The photocatalytic H_2 evolution was executed from an aqueous methanol solution (MeOH:H_2O = 1:3 v/v) in a closed 37 mL quartz cell. Reaction conditions remained identical: the amount of photocatalyst at 10 mg/10 mL, room temperature, and testing duration of five hours under UV-visible irradiation triggered by a Mercury lamp (150W). These cells were degassed by Argon for 20 min to remove dissolved oxygen completely before illumination.

To compare the performance of our systems with the reported studies, we employed ICP-OES (Inductively Coupled Plasma Optical Emission Spectrometry) to quantify the mass percentage of either elements (Si, Ti, O, Metal nanoparticles) or oxides (SiO_2, TiO_2, metal NPs) in our powder samples. Particularly, the insulting SiO_2 occupies approximately 85 wt% and the remaining active phase (TiO_2 and metal nanoparticles) accounts for 15 wt%. In each photocatalytic test we used 10 mg of photocatalyst powder. As the silica core has no effect on the kinetic of photocatalytic reaction as demonstrated by new experiments, the initial H_2 evolution rate was normalized by the amount of active phase, (15 wt%).

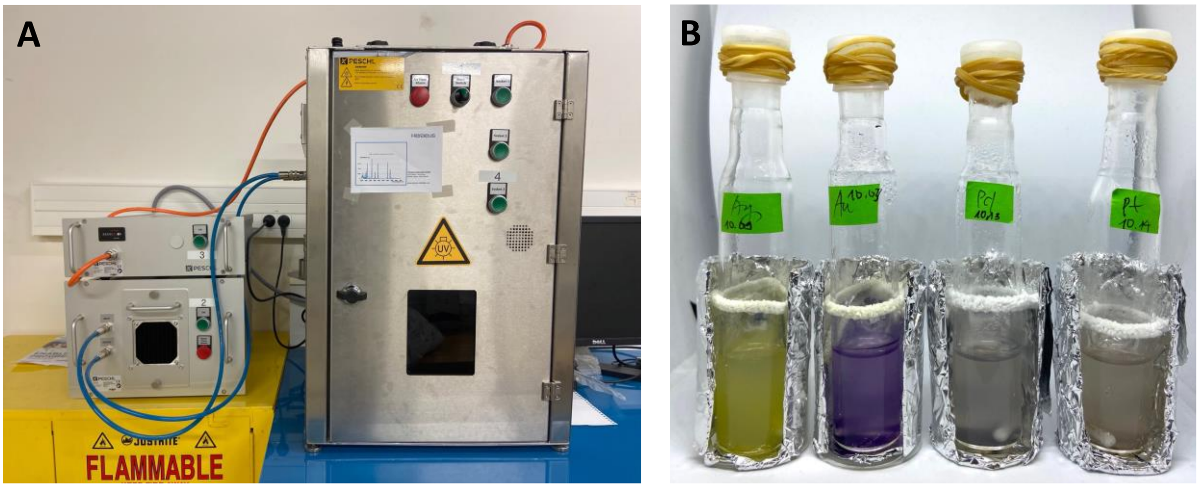

Figure S15| (**A**) Ultraviolet-visible photoreactor. (**B**) Photocells, containing reaction solution of SMSI-like core@shell photocatalysts, after 5 hours of irradiation.

Liquid Chromatography

Liquid phase products, including HCHO and CH_3CHO, were detected by high-performance liquid chromatography (HPLC) on Agilent 1260 Infinity with UV-Vis detector. We followed a derivatization method[11], which is based on the trace detection of the complex between 2,4-dinitrophenylhydrazine (DNPH) and analytes containing carbonyl group (-C=O) as following:

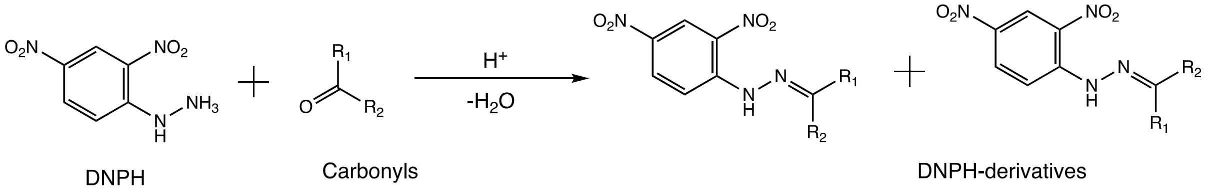

For photocatalytic H_2 evolution reactions, a HPLC vial contains extracted reaction solution (1 mL), H_3PO_4 5N (40 µL), DNPH in acetonitrile 1mg/mL (400 µL), and acetonitrile (1 mL). After sonicating 15 minutes, the solution was injected to on-site HPLC instrument. Acetonitrile (CH_3CN) and deionized water (H_2O) were used as the eluent in a reverse phase analytical column. Signals were recorded by an UV-visible detector at wavelength of 360 nm.

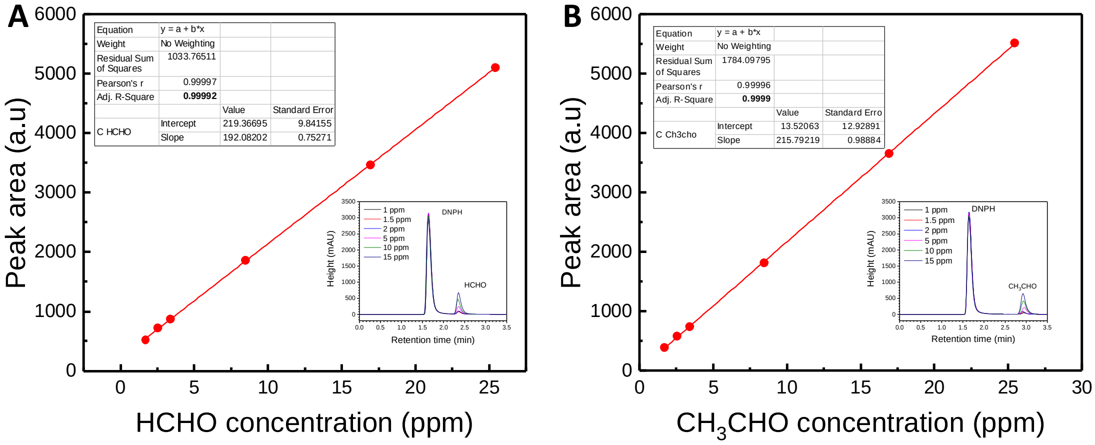

Figure S16| (**A**) Ultraviolet-visible photoreactor. (**B**) Photocells, containing reaction solution of SMSI-like core@shell photocatalysts, after 5 hours of irradiation.

In order to determine the linear range of the HCHO and CH_3CHO measurements, two series of HCHO and CH_3CHO solution having concentration from 1 ppm to 15 ppm were prepared. The obtained correlation coefficient (R^2) at 0.9999 of both analytes were higher than 0.99 as required by AOAC for correlation factor of calibration curves.[12] This evidenced the high linearity between the measurement signal and the concentration of analyte, which is a requirement for high precision of measurement result. Based on our calibration curves, the limit of detection (LOD) and the limit of quantitation (LOQ) were calculated as following:

$$Y_L = y_b + k \times S_b$$

where Y_L is average signal obtained from blank samples after experiments (at least 10 independent experiments), S_b is standard deviation for the repeated measurement of the blank signal, and k is arithmetic quantity chosen according to the expected confidence. Therefore,

$$X_L = X_b + \frac{k \times S_b}{b}$$

In the blank sample, $X_b = 0$ and $k = 3$, leading to:

$$LOD = \frac{3 \times S_b}{b}$$

$$LOQ = \frac{10 \times S_b}{b}$$

where S_b is error fo value y in the regression equation and b is linear regression coefficient. The values of LOD and LOQ were noted in the **table S2**:

Table S2| LOD and LOQ values of HCHO and CH_3CHO analytical procedures.

Analytes	*LOD (ppm)*	*LOQ (ppm)*
HCHO	0.22457725	0.74859082
CH_3CHO	0.26261214	0.87537381

References.

[1] W. Stöber, A. Fink, E. Bohn, *J. Colloid Interface Sci.* **1968**, *26*, 62–69.

[2] A. Heuer-Jungemann, N. Feliu, I. Bakaimi, M. Hamaly, A. Alkilany, I. Chakraborty, A. Masood, M. F. Casula, A. Kostopoulou, E. Oh, K. Susumu, M. H. Stewart, I. L. Medintz, E. Stratakis, W. J. Parak, A. G. Kanaras, *Chem. Rev.***2019**, *119*, 4819–4880.

[3] D. G. Duff, A. Baiker, P. P. Edwards, *Langmuir* **1993**, *9*, 2301–2309.

[4] G. D. Gesesse, C. Wang, B. K. Chang, S.-H. Tai, P. Beaunier, R. Wojcieszak, H. Remita, C. Colbeau-Justin, M. N. Ghazzal, *Nanoscale* **2020**, *12*, 7011–7023.

[5] M. N. Ghazzal, H. Kebaili, M. Joseph, D. P. Debecker, P. Eloy, J. De Coninck, E. M. Gaigneaux, *Appl. Catal., B* **2012**, *115–116*, 276–284.

[6] M. N. Ghazzal, D. P. Debecker, E. M. Gaigneaux, *Thin Solid Films* **2016**, *611*, 117–124.

[7] J. Tauc, R. Grigorovici, A. Vancu, *Phys. Status Solidi B* **1966**, *15*, 627–637.

[8] D. Reyes-Coronado, G. Rodríguez-Gattorno, M. E. Espinosa-Pesqueira, C. Cab, R. de Coss, G. Oskam, *Nanotechnology* **2008**, *19*, 145605.

[9] P. Kubelka, F. Munk, *A Contribution to the Optics of Pigments*, Z. Technol. Phys, **1931**.

[10] D. M. King, X. Du, A. S. Cavanagh, A. W. Weimer, *Nanotechnology* **2008**, *19*, 2–7.

[11] A. Soman, Y. Qiu, L. Q. Chan, *J. Chromatogr. Sci.* **2008**, *46*, 461–465.

[12] AOAC International, *AOAC International and Official Method of Analysis* **2016**, 1–18.
